# Long‐term elevated precipitation promotes an acid metabolic preference in soil microbial communities in a Tibetan alpine grassland

**DOI:** 10.1128/msystems.00470-25

**Published:** 2025-07-28

**Authors:** Xiaomin Fan, Xue Guo, Qi Qi, Haoran Gui, Yujiang Li, Yunfeng Yang, Jin-Sheng He, Linwei Wu

**Affiliations:** 1 Institute of Ecology, Key Laboratory for Earth Surface Processes of the Ministry of Education, College of Urban and Environmental Sciences, Peking University 105876, Beijing, China; 2 State Key Laboratory of Urban and Regional Ecology, Research Center for Eco-Environmental Sciences, Chinese Academy of Sciences, Beijing, China; 3 Department of Microbiology and Environmental Systems Science, Division of Microbial Ecology, University of Vienna 115351, Vienna, Austria; 4 State Key Laboratory of Herbage Improvement and Grassland Agro-Ecosystems, College of Pastoral Agriculture Science and Technology, Lanzhou University 467666 , Lanzhou, China; 5 Institute of Environment and Ecology, Tsinghua Shenzhen International Graduate School, Tsinghua University 12442, Shenzhen, China; E O Lawrence Berkeley National Laboratory, Berkeley, California, USA

**Keywords:** climate change, active soil microbes, carbon metabolic preferences, greenhouse gas emissions, alpine ecosystems, metatranscriptomics

## Abstract

Alpine ecosystems store vast amounts of soil organic carbon but are highly sensitive to climate change. Despite this, the response of *in situ* soil microbial metabolic processes, especially carbon substrate utilization, to climatic shifts remains underexplored. Here, we assessed microbial activity by metatranscriptomics in a Tibetan alpine grassland after a decade of experimental warming (+2°C) and altered precipitation (+50% and −50% of ambient precipitation). The experiment revealed that altered precipitation, rather than warming, shaped the active microbial community. Altered precipitation and warming had significant interactions: warming combined with increased precipitation generally suppressed microbial carbohydrate metabolism and methane oxidation, while warming with decreased precipitation enhanced these processes. Notably, increased precipitation induced a shift in microbial communities towards acid metabolism over sugar metabolism, predominantly driven by taxa such as Betaproteobacteria. This metabolic shift corresponded with an increased emission ratio of methane (CH_4_) to carbon dioxide (CO_2_), a change primarily driven by CH_4_, underscoring the critical role of microbial carbon metabolic preferences in regulating greenhouse gas emissions. Our findings highlight the necessity of integrating microbial carbon metabolic preferences and their interactions with climatic factors into models to accurately predict carbon-climate feedbacks.

**IMPORTANCE:**

Microbes have specific preferences for different carbon substrates, but their responses to climate change remain unclear. Our study, conducted through a long-term climate manipulation experiment in a Tibetan alpine grassland, reveals that increased precipitation leads soil microbial communities to favor acid metabolism over sugar metabolism. This shift significantly affects greenhouse gas emissions by increasing the CH_4_/CO_2_ ratio, which has important implications for global warming. These findings are crucial for accurately forecasting carbon-climate feedbacks and managing alpine ecosystems as climate change progresses.

## INTRODUCTION

The Tibetan Plateau, Earth’s largest and highest highland area, stores substantial soil organic carbon (SOC) and represents a key component of the global carbon cycle (1, 2). This region is experiencing accelerated warming and shifting precipitation patterns, making it a critical area for investigating carbon-climate feedbacks (1, 3). Given that soil microbes are key regulators of SOC mineralization (4), the accelerated warming poses a particular risk of increasing microbial decomposition of SOC, thereby enhancing the release of greenhouse gases like carbon dioxide (CO_2_) and methane (CH_4_), which contributes to a positive carbon-climate feedback (5, 6). Additionally, changes in precipitation patterns can influence microbial carbon metabolism by altering soil water availability, which affects physiological stress, resource availability, and substrate quality (4, 7, 8). These interactions complicate predictions of soil carbon feedback to climate change. Currently, the mechanisms driving the carbon-climate feedback remain one of the greatest uncertainties in Earth system models forecasting future climate warming (9–12). This uncertainty is partly due to a limited mechanistic understanding of how microbial metabolic processes, particularly carbon substrate utilization, respond to climatic shifts *in situ*. Understanding these microbial responses is essential for improving projections of carbon dynamics under future climate scenarios.

Field experiments on climate change have shown that warming could either increase (13, 14) or decrease (15) the potential for microbial carbon metabolism in soils, as indicated by metagenomic or functional gene array methods. Moreover, these DNA-based methods primarily reflect the potential functional capacity of microbial communities, not their actual metabolic activity. In contrast, metatranscriptomics can provide direct evidence of gene expression related to enzyme production (16). Laboratory incubation studies using metatranscriptomics have demonstrated that elevated temperatures and reduced moisture levels can shift active microbial communities and functional gene expressions, leading to increased CO_2_ and CH_4_ production (17, 18). Despite these findings, the relevance of laboratory results to natural settings remains speculative, as lab conditions fail to replicate the complexity of *in situ* environments (19, 20). Therefore, understanding the active microbial communities and their functions in long-term field experiments is crucial for predicting greenhouse gas fluxes and assessing ecosystem responses to climate change more accurately.

Microbes in soil utilize various carbon substrates through specific enzymes, such as carbohydrate-active enzymes, involved in carbon metabolism (15, 21, 22). Recent cultivation-based studies suggest that the catabolic niches of heterotrophic bacteria can be primarily partitioned by their substrate preferences for sugars or acids (22, 23). This raises the question of how climate change might alter microbial carbon metabolic preferences in soil. Furthermore, the type and quality of carbon substrates in the soil significantly impact CO_2_ and CH_4_ emissions (24, 25). For instance, the presence of more labile carbon substrates in soil has been associated with increased apparent activation energies for CH_4_ relative to CO_2_, suggesting a shift toward higher CH_4_ emissions (26). Further, CH_4_ flux is regulated by microbial-mediated methanogenesis and methanotrophy (27, 28). Methanogens, primarily utilizing H_2_ and CO_2_, acetate, and methylated compounds to form CH_4_, primarily belong to archaeal lineages within the Euryarchaeota, such as the orders *Methanobacteriales* and *Methanomicrobiales*, while methanotrophs include both methanotrophic archaea and bacteria, such as the genera *Candidatus Methanoperedens* and *Methylococcus* (28, 29). However, few studies have investigated the relationship between soil microbial carbon metabolic preferences and CH_4_ metabolism. Given that the global warming potential of CH_4_ from non-fossil sources is 27 times that of CO_2_ on a 100-year timescale, understanding the ratio of CH_4_ to CO_2_ emissions (CH_4_/CO_2_) is crucial for assessing the magnitude of carbon-climate feedback (12, 30). Thus, it is essential to investigate the connections between microbial carbon metabolic preferences and the relative emissions of CH_4_ and CO_2_ under changing climatic conditions.

To address these issues, a field climate manipulation experiment was established in 2011 in a Tibetan alpine grassland. Using a randomized block design, warming (+2°C) and altered precipitation treatments (−50% ambient precipitation as drought, +50% as wet) were applied continuously for 10 years. Six experimental blocks were established, each containing six treatment plots: ambient (control), warming, drought, wet, warming+drought, and warming+wet. In this study, we collected soil samples in August 2020 for metatranscriptome sequencing and measured carbon fluxes *in situ*, aiming to assess the responses of active soil microbes to long-term warming and altered precipitation. We explore the following major issues: how long-term climatic changes influence microbial transcripts involved in carbon metabolism; how microbial carbon metabolic preferences of sugar versus acid are affected; and how these microbial metabolic processes are linked to CO_2_ and CH_4_ fluxes. Based on metagenomic evidence demonstrating temperature-driven shifts in microbial metabolic potential (14, 31), we hypothesize that warming may increase microbial transcripts associated with carbon metabolism. Furthermore, given that water addition could enhance the microbial gene abundance associated with amino acid degradation while reducing that associated with sugars (32), we hypothesize that microbial communities may preferentially utilize acids in wetter soils.

## MATERIALS AND METHODS

### Study site and soil sampling

The experiment site is located at the Haibei Alpine Grassland Ecosystem Research Station on the northeastern Tibetan Plateau in Qinghai Province, China (101°12′E, 37°37′N; elevation 3200 m). This region experiences a continental monsoon climate, with a mean annual temperature of −1.1°C and average annual precipitation of 485 mm. The perennial plants, such as *S. aliena* and *E. nutans*, are dominant species in this ecosystem. The soil type at this site is Mat-Gryic Cambisol, with the surface soil (0–10 cm) having an average pH of 6.4 (33).

The detailed experimental design has been described previously (31, 33). In brief, the long-term *in situ* climate manipulation experiment was established in 2011 with a randomized block design in which altered precipitation (ambient precipitation, 50% decrease of ambient precipitation, and 50% increase of ambient precipitation) and warming (ambient temperature, increased soil temperature by 2 ^o^C in the top 5 cm layer) were treatment factors. This experimental site contained six experimental blocks, and each block comprised six treatment plots, resulting in a total of 36 plots, each measuring 1.8 m × 2.2 m. For each warming plot, two 1,200 W infrared heaters, each measuring 1,000 mm in length and 22 mm in width, were suspended in parallel 150 cm above the ground. To control the received precipitation in each plot, rain shelters composed of four “V”-shaped transparent polycarbonate resin channels were fixed at a 15° angle above the infrared heaters. We manually redistributed the collected rainfall from the decreased precipitation plots to the increased precipitation plots following each precipitation event using a spray bottle. In the ambient plots, two “dummy” infrared heaters and four “dummy” transparent polycarbonate resin channels were installed to reduce the effects of shading.

Soil samples were collected at approximately the time of peak plant biomass, mid-August 2020. In each plot, eight topsoil cores (0–10 cm) were randomly collected and combined into a composite sample through a 2 mm mesh to remove stones and root fragments in the field immediately. Then, a 2 g composite sample was collected in 50 mL sterile centrifuge tubes. Approximately 5 mL of LifeGuard Soil Preservation Solution (Qiagen, Hilden, Germany) was added to soak the soil samples, and the tubes were sealed. The soil samples were then transported to the laboratory at low temperature for RNA extraction within 24 hours. The remaining fresh soil samples were also transported to the laboratory for soil chemical measurements.

### Soil properties determination

All measurements of carbon fluxes and soil properties were conducted in 2020. The EM50 data loggers (Meter, Inc.) equipped with 5-TM probe sensors were used to automatically monitor soil temperature and moisture hourly at the soil depth of 5 cm. To match the soil sampling data, the mean temperature and moisture in August were calculated for further analysis.

Soil pH was measured using the potentiometric method. Briefly, 5 g of fresh soil sample was weighed, and 25 mL of distilled water was added. The mixture was shaken at 180 r/min for 30 minutes, then allowed to settle before measuring the pH using a pH meter. Soil microbial biomass carbon was determined using the chloroform fumigation-extraction method. In brief, 5 g fresh soil was fumigated with ethanol-free CHCl_3_ for 24 hours. Then, both the fumigated and 5 g unfumigated soil samples were extracted with 20 mL 0.5 mol/L K_2_SO_4_ (solution:soil = 4:1) by shaking at 180 rpm for 30 minutes. After that, the contents were filtered through a medium-speed quantitative filter paper and a 0.45 µm membrane filter. The organic carbon in the filtered solution was then measured using a TOC/TN analyzer. Finally, soil microbial biomass carbon was determined by measuring the difference in organic carbon concentration between the fumigated and unfumigated samples.

### Carbon fluxes measurement

In August 2020, soil CO_2_ and CH_4_ fluxes were measured using the trace gas analyzer LI-7810 (CH_4_/CO_2_/H_2_O, LI-COR Biosciences, Lincoln, USA) between 9:00 am and 11:00 am on sunny days. PVC collars were installed in the plots to measure soil carbon fluxes, including soil respiration, soil heterotrophic respiration, and CH_4_ flux. PVC collars with a diameter of 20 cm and a height of 8 cm were inserted 3 cm into the ground to measure soil respiration. Heterotrophic respiration was determined using PVC collars with a diameter of 20 cm and a height of 70 cm, which were inserted 65 cm into the ground. To accurately measure heterotrophic respiration, aboveground plants within the 70 cm collars were frequently clipped to cause all plants inside the collars to die. Soil CH_4_ flux was also monitored using the LI-7810 in the PVC collars. To investigate changes in soil carbon fluxes related solely to soil microbes and their relationships, this study used CH_4_ flux from the 70 cm collars and calculated the emission ratio of CH_4_ to heterotrophic respiration (CO_2_).

Ecosystem carbon fluxes were measured using the transparent chamber method between 9:00 am and 12:00 pm on sunny days. In brief, an infrared gas analyzer LI-6400 (LI-COR Biosciences, Lincoln, USA) was connected to a transparent chamber with dimensions of 0.4 m × 0.4 m × 0.6 m to measure ecosystem respiration and net ecosystem exchange under shaded and unshaded conditions, respectively.

### RNA extraction, sequencing, and bioinformatic processing

The total RNA was extracted from 2 g of soil using the RNeasy PowerSoil Total RNA kit (QIAGEN) following the manufacturer’s instructions. We extracted a total of 36 soil samples and ultimately obtained 32 qualified RNA. The qualified RNA was then sent for library preparation. After that, RNASeq libraries were sequenced on an Illumina platform (2 × 150 bp paired-end Illumina MiSeq).

Metatranscriptome sequencing of 32 soil samples yielded 354.6 Gb of raw data. The low-quality reads and sequencing adapters were trimmed using BBDuk v38.18 with the parameters “ktrim = r k = 23 mink = 11 hdist = 1 qtrim = r trimq = 20 maq = 20”. The quality of resulting reads was checked using FastQC v0.11.9. The quality-controlled reads were further sorted into rRNA and non-rRNA sequences using SortMeRNA v 2.0 with reference databases, including SILVA archaeal 16S and 23S rRNA, bacterial 16S and 23S rRNA, eukaryotic 18S and 28S rRNA, and Rfam for 5S and 5.8S (34). The non-rRNA fraction from each sample was *de novo* assembled using Megahit v1.2.9 with the parameters “--k-list 31,51,71,91,111,131 --kmin-1pass --min-contig-len 200” (35). Transcripts were predicted for each assembled contig using Prodigal v2.6.3 (36), and the representative transcripts were clustered using MMseqs2 v13.45111 with the parameters “--min-seq-id 0.90 c 0.9” (37). The abundance of each representative transcript in each sample was calculated using RSEM by mapping the quality-controlled non-rRNA reads against the representative transcripts (38). The abundance profiles were normalized to obtain the relative abundance matrix, measured in counts per million (CPM), using the TMM method in edgeR (39).

Taxonomic assignment of the representative transcripts was performed using blastp in Diamond v0.9.14.115 against the clustering NCBI-nr database (https://github.com/Arcadia-Science/2023-nr-clustering) with the parameters “--sensitive -e 0.00001” (40). CAZy, including glycoside hydrolases, glycosyltransferases, polysaccharide lyases, carbohydrate esterases, auxiliary activities, and carbohydrate binding, was annotated using run_dbcan (41). Transcripts associated with methane metabolism were predicted against the KEGG database using kofamScan v1.3.0 with an *E*-value cutoff of 0.00001 (42). *mcr* transcripts were specifically annotated with two databases (KEGG and MCycDB) (43). To calculate the carbon substrate metabolic preferences of soil microbes, transcripts associated with the transporter and catabolism of acids and sugars were annotated using the Transporter Classification Database (TCDB) and eggNOG, respectively (44, 45). Transcripts associated with membrane transport proteins were annotated using blastp in Diamond against TCDB. Referring to a previous study (23), we filtered the corresponding membrane transport proteins for sugar and acid substrates, including the isoforms of these transport proteins. EggNOG was annotated using Diamond mode with default parameters, and the annotated transcripts were classified into acid or sugar catabolism based on a previous study (23). The sugar-to-acid (SAR) ratio was defined as the ratio of the relative abundance of transcripts involved in sugar transporters or catabolism to the relative abundance of transcripts involved in acid transporters or catabolism. SAR-Transporter represented the transcripts associated with carbon substrate transport, while SAR-Catabolism represented the transcripts associated with carbon substrate catabolism.

### Statistical analyses

All statistical analyses were conducted using R software 4.2.1 (https://www.r-project.org). Considering that the experimental data were not independent due to experimental design, LMMs implemented using the “lme4” R package (46) were employed to evaluate the experimental treatment effects on environmental factors, carbon fluxes, and the relative abundance of transcripts, with the model specified as *y* ~ altered precipitation × warming + (1|block). In these LMMs, fixed effects were altered precipitation (coded as 0 for ambient precipitation, −0.5 for decreased precipitation, and 0.5 for increased precipitation), warming (coded as 0 for ambient temperature, and 1 for increased temperature), and their interaction, while block was treated as a random effect. The relative abundance of transcripts was rescaled to have a mean of 0 and a standard deviation of 1 for the purposes of statistical analysis. The regression coefficients in the LMMs represented the effect sizes, while the effect sizes of drought and wet were the effect sizes of altered precipitation multiplied by −0.5 and 0.5, respectively. For example, the effect size of altered precipitation on soil moisture was 0.09, meaning that the effect size of drought on soil moisture was −0.045 (i.e., a 50% decrease in precipitation coupled with a 4.5% decrease in moisture).

The correlations between environmental factors, carbon fluxes, and microbial transcripts were analyzed using LMMs, specified as microbial transcript ~environmental factor + (1|block), carbon flux ~microbial transcript + (1|block), and CH_4_/CO_2_ ~ soil moisture + soil temperature + SAR + CAZy + (1|block). The variance explained by the fixed effect in LMMs was determined by marginal coefficient of determination using the ‘r.squaredGLMM’ function from the “MuMIn” R package (47). The effect sizes of multiple variables on CH_4_/CO_2_ in multiple regression analysis were calculated by utilizing standardized data. Statistical significance (*P*) was determined by Type II Wald χ tests using the “car” R package (48). Difference in transcript composition under different treatments was visualized by principal coordinate analysis (PCoA) based on the weighted Bray-Curtis distance using the ‘pcoa’ function in “ape” R package (49), and statistical significance was assessed by PERMANOVA using the “Adonis” function in “vegan” R package (50). All figures in this study were generated using R software.

## RESULTS

### Effects of climate change on soil variables and carbon fluxes

**Fig 1:**
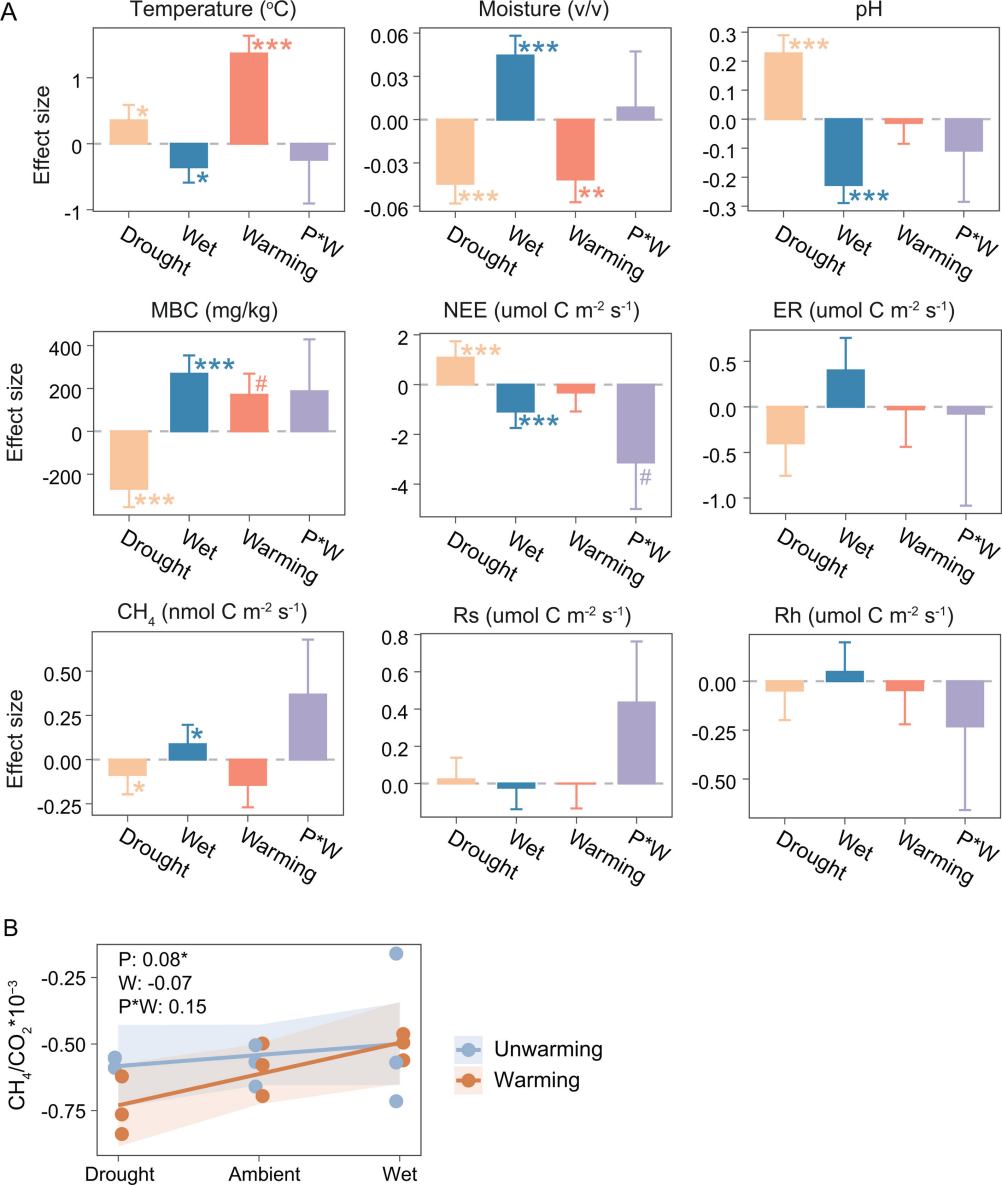
Effects of experimental treatments on soil variables and carbon fluxes. (**A**) Effects of altered precipitation and warming on environmental factors, microbial biomass carbon, and carbon fluxes. (**B**) Effects of altered precipitation and warming on CH_4_/CO_2_. The effect sizes were regression coefficients in the LMMs [LMMs: *y* ~ altered precipitation*warming + (1|block)]. The effect sizes of drought and wet were the effect sizes of altered precipitation multiplied by −0.5 and 0.5, respectively. Data were coefficients and standard errors of LMMs. Type II Wald χ tests were used to determine statistical significance: ***P < 0.001, **P < 0.01, *P < 0.05, ^#^ *P* < 0.1. Abbreviations in subplot: P, altered precipitation; W, warming; P*W, interaction between altered precipitation and warming. Data in the corner of plot B were regression coefficients (β) in the LMMs. Environmental factors: soil temperature, soil moisture, pH. Microbial biomass carbon: MBC. Ecosystem carbon fluxes: NEE (net ecosystem carbon exchange), ER (ecosystem respiration). Soil carbon fluxes: CH_4_, Rs (soil respiration), Rh (heterotrophic respiration).

Linear mixed-effects models (LMMs) were employed to quantify the effects of experimental warming and precipitation alteration on soil variables and carbon fluxes. The regression coefficients of the LMMs, representing the effect sizes (β), indicate the direction and magnitude of the experimental treatment effects. Warming significantly increased soil temperature by 1.37℃ (β = 1.37, *P* = 4.58E-07) and decreased soil moisture by 4% (β = −0.04, *P* = 0.009) (Fig. 1A; Table S1). Changes in precipitation were significantly correlated with soil moisture (β = 0.09, *P* = 1.73E-06), i.e., half precipitation decreased soil moisture by 4.5%. Altered precipitation also changed soil temperature (β = −0.71, *P* = 0.012), pH (β = −0.45, *P* = 9.21E-09), and soil microbial biomass carbon (MBC) (β = 536.03, *P* = 2.04E-07). Additionally, warming had a marginal positive effect on MBC (β = 169.88, *P* = 0.086).

Regarding the soil carbon fluxes, LMMs indicated that the precipitation level positively affected CH_4_ (β = 0.17, *P* = 0.022), but not soil respiration (β = −0.04, *P* = 0.292 on soil total respiration; β = 0.09, *P* = 0.924 on heterotrophic respiration) (Fig. 1A; Table S1). Consequently, the relative emissions of CH_4_ and CO_2_, calculated by the ratio of CH_4_ to heterotrophic respiration fluxes, significantly increased with the precipitation level (β = 0.08, *P* = 0.019) (Fig. 1B; Table S1). In comparison, warming did not affect these soil carbon fluxes. Although the interaction effects of altered precipitation and warming on soil carbon fluxes were statistically insignificant, the effect sizes were substantial. Net ecosystem exchange exhibited a negative correlation with the precipitation level (β = −2.16, *P* = 6.79E−05), and warming further amplified this negative correlation (interaction β = −3.13, *P* = 0.095). In summary, both warming and altered precipitation significantly changed the soil microclimate, while altered precipitation had predominant effects on soil carbon fluxes.

### Active soil microbes and their responses to climate change

**Fig 2:**
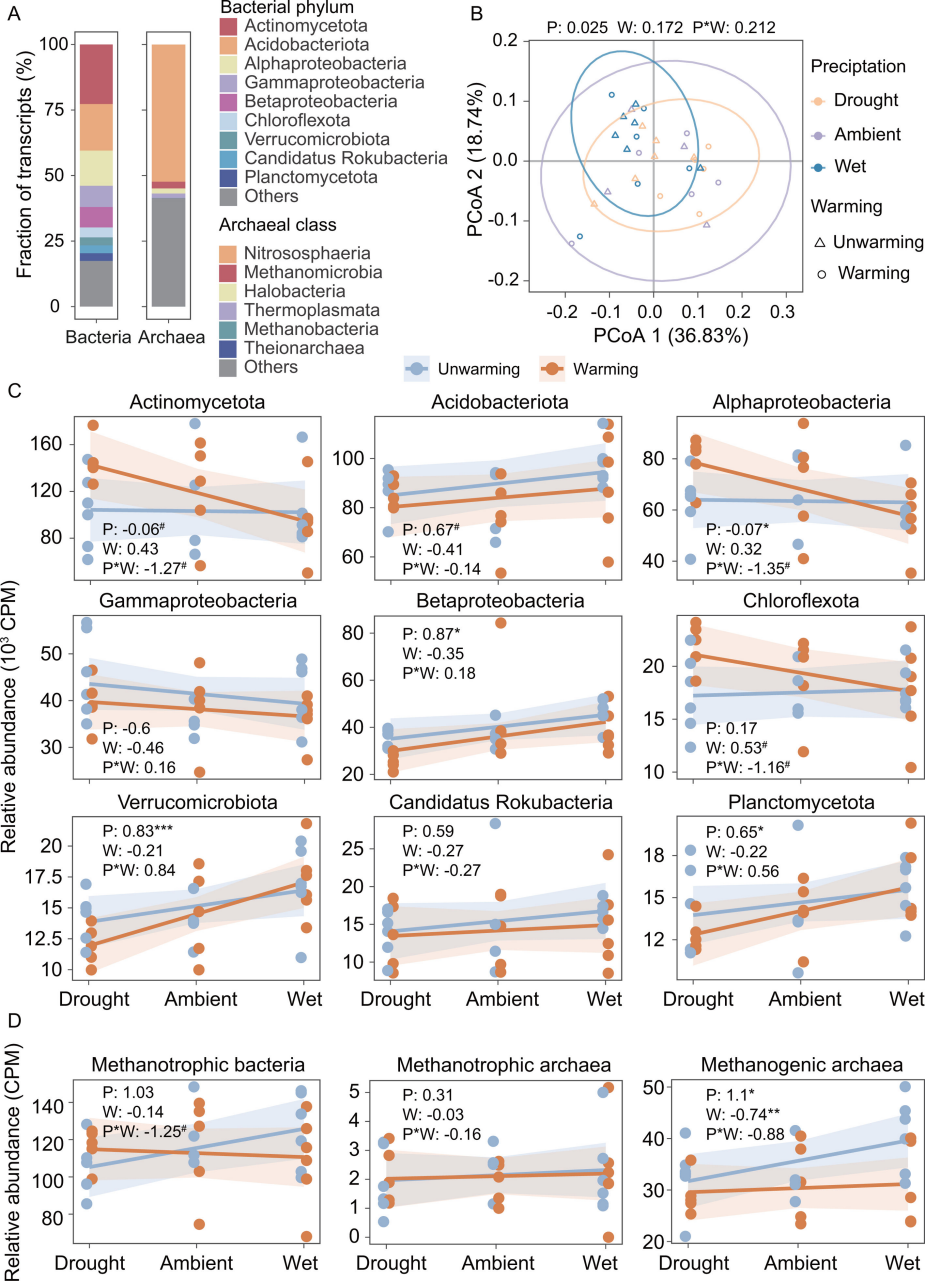
Effects of experimental treatments on active soil microbes. (**A**) Proportion of the relative abundance of phylum-level affiliations of bacterial transcripts and class-level affiliations of archaeal transcripts across all plots. Pseudominadota was divided into Alphaproteobacteria, Betaproteobacteria, and Gammaproteobacteria based on the class level. (**B**) Difference in active soil microbial composition. The figure showed the first and second axes of principal coordinate analysis (PCoA) based on the weighted Bray-Curtis distance of the relative abundance of species-level transcripts. The values in parentheses on the axes represented the relative eigenvalues of PCoA. The values at the top of the subplot indicated the statistical significance (*P* values) determined by PERMANOVA using Adonis. (**C**) Relative abundance of taxonomic affiliations of transcripts associated with bacteria. (**D**) Relative abundance of taxonomic affiliations of transcripts associated with methanotrophs and methanogens. The values in the corner of each subplot represented the effect sizes of treatments on them [LMMs: relative abundance ~altered precipitation*warming + (1|block)]. Type II Wald χ tests were used to determine statistical significance: ***P < 0.001, **P < 0.01, *P < 0.05, ^#^ *P* < 0.1. Abbreviations in subplots: P, altered precipitation; W, warming; P*W, interaction between altered precipitation and warming.

To identify the active soil microbes, we analyzed the taxonomic affiliations of transcripts. A total of 94.14% of the transcripts were affiliated with bacteria (Table S2), which were predominantly from Actinomycetota (22.73% of bacterial transcripts), Acidobacteriota (17.76%), Alphaproteobacteria (13.43%), Gammaproteobacteria (8.09%), and Betaproteobacteria (7.81%) (Fig. 2A; Table S3). In comparison, 0.28% of the transcripts were derived from archaea, mainly belonged to Nitrososphaeria (52.33% of archaeal transcripts), Methanomicrobia (2.56%), and Halabacteria (1.95%) (Fig. 2A; Table S4). Among the active microbial community, the most abundant bacterial species were *Lacunisphaera limnophila*, *Staphylococcus aureus*, and *Pseudonocardia* sp., while *Nitrosopumilus* sp., *Candidatus Nitrosocosmicus arcticus*, and *Candidatus Nitrosocosmicus oleophilus* were the most abundant archaeal species.

PERMANOVA analysis demonstrated that altered precipitation significantly impacted the composition of active soil microbes (*P* = 0.025), although the microbial community structure was not clearly separated in the two-dimensional ordinations. In contrast, warming (*P* = 0.172) and its interaction with altered precipitation (*P* = 0.212) did not have significant effects (Fig. 2B). LMMs were further utilized to examine the treatment effects on different microbial lineages. The relative abundance of transcripts from Acidobacteriota, Betaproteobacteria, Verrucomicrobiota, and Planctomycetota increased with the precipitation level (β = 0.65-0.87 on rescaled abundances, *P* < 0.1), independent of warming (Fig. 2C; Table S5). In contrast, negative interaction effects of the precipitation level and warming were observed for Actinomycetota, Alphaproteobacteria, and Chloroflexota; that is, their abundance increased in warmer and drier soils but declined in warmer and wetter soils (Fig. 2C; Note SA).

We also examined the treatment effects on functional guilds related to methane metabolism, based on the known microbial lineages of methanotrophs and methanogens. Our analysis indicated a marginally significant interaction effect of the precipitation level and warming on methanotrophic bacteria (β = −1.25, *P* = 0.061), showing that methanotrophic bacteria abundance increased with the precipitation level under ambient temperature, but declined with the precipitation level in warmer soils (Fig. 2D; Table S6). In comparison, the abundance of methanotrophic archaea was not affected by warming or precipitation alteration. Methanogenic archaea significantly increased with the precipitation level (β = 1.10, *P* = 0.048) and decreased with warming (β = −0.74, *P* = 9.99E-03). Further, different lineages within a functional guild could respond differentially to the treatments. For instance, Methanobacteriales positively correlated with the precipitation level (β = 0.74, *P* = 0.020), while Methanomicrobiales was negatively impacted by warming (β = −0.83, *P* = 0.007) (Fig. S1; Note SA). These results suggest that warming and precipitation alteration have differential impacts on various microbial lineages and functional guilds, and their interactions are significant on various active microbes.

### Impacts of climate change on microbial carbohydrate and methane metabolism

**Fig 3:**
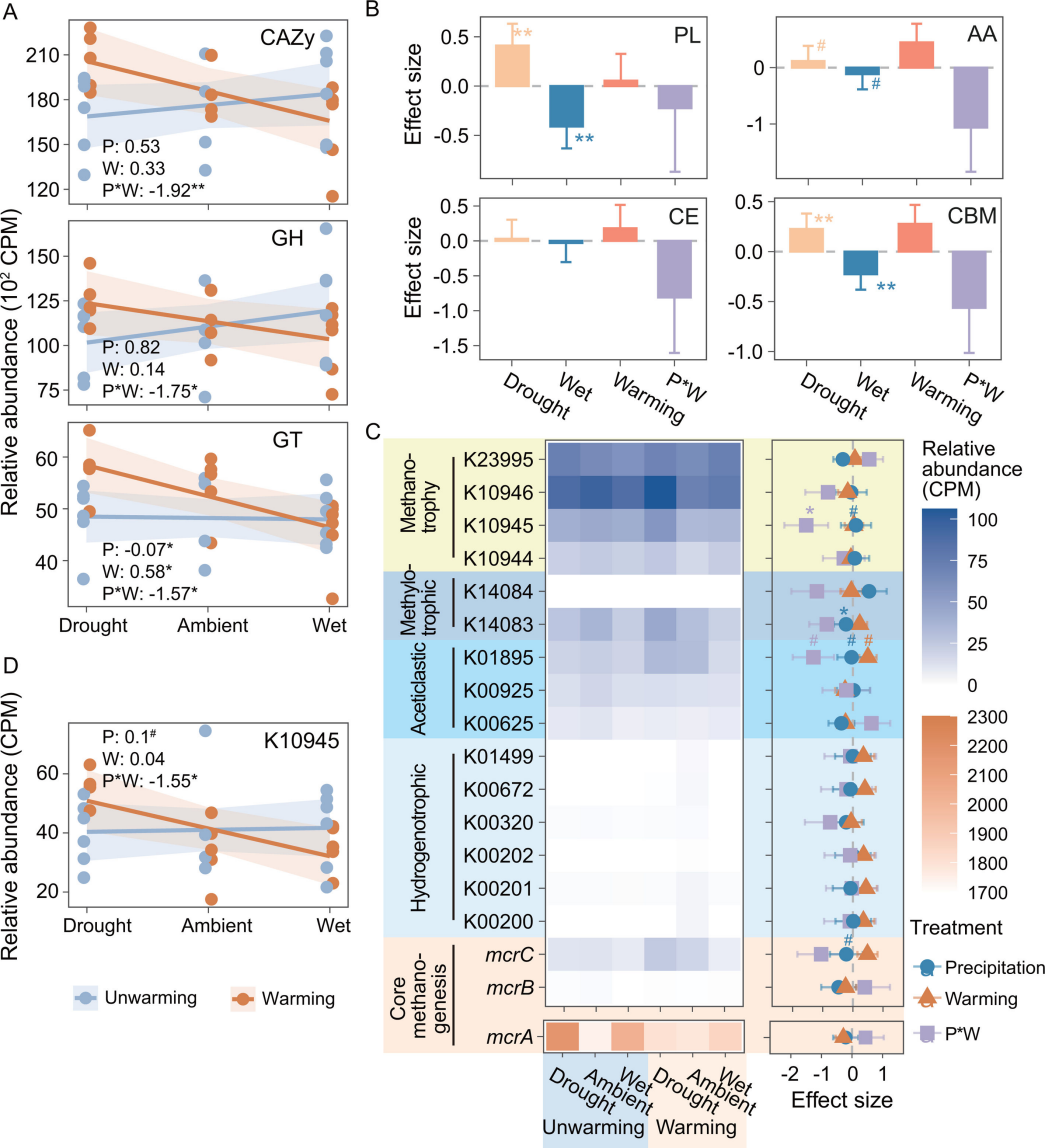
Effects of experimental treatments on microbial carbohydrate and methane metabolism. (**A**) Relative abundance of transcripts associated with CAZy and its modules, i.e., glycoside hydrolases (GH) and glycosyltransferases (GT). (**B**) Effects of altered precipitation and warming on the relative abundance of transcripts associated with polysaccharide lyases (PL), auxiliary activities (AA), carbohydrate esterases (CE), and carbohydrate binding (CBM). (**C**) Relative abundance of transcripts associated with methane metabolism across drought, ambient, wet, drought and warming, warming, as well as wet and warming plots (left). Effects of altered precipitation and warming on the relative abundance of transcripts associated with methane metabolism (right). Data were coefficients and standard errors of LMMs. Methane metabolism included methanotrophy (four gene families) and methanogenesis (14 gene families), which was divided into methylotrophic methanogenesis (two gene families), aceticlastic methanogenesis (three gene families), and hydrogenotrophic methanogenesis (six gene families) based on the substrates and core methanogenesis (three gene families). (**D**) Relative abundance of transcripts associated with K10945 (*pmoB-amoB*). The values in the corner of each subplot represented the effect sizes of experimental treatments on them [LMMs: relative abundance ~altered precipitation*warming + (1|block)]. Type II Wald χ tests were used to determine statistical significance: **P < 0.01, *P < 0.05, ^#^*P* < 0.1. Abbreviations in subplots: P, altered precipitation; W, warming; P*W, interaction between altered precipitation and warming.

To understand the response of microbial carbohydrate metabolism to climate change, we annotated the transcripts to the Carbohydrate-Active enZYmes (CAZy) database (51). Inconsistent with our hypothesis, there was a significant interaction effect of the precipitation level and warming on the relative abundance of transcripts associated with CAZy (β = −1.92, *P* = 0.007), i.e., total CAZy abundance increased (21.22%) in warmer and drier soils, but declined (3.40%) in warmer and wetter soils compared to ambient temperature and precipitation (Fig. 3A; Table S8). Similar patterns were observed for transcripts involved in glycoside hydrolases and glycosyltransferases (Fig. 3A and Note SB1). The relative abundance of transcripts associated with polysaccharide lyases, auxiliary activities, and carbohydrate binding significantly or marginally significantly decreased with the precipitation level (β = −0.82 to −0.23, *P* < 0.1), independent of the warming treatment (Fig. 3B; Table S8).

We also examined microbial gene expressions associated with methane metabolism, including methanotrophy and methanogenesis, by annotating Kyoto Encyclopedia of Genes and Genomes (KEGG) Orthology. Our data revealed the expression of eighteen gene families involved in methane metabolism, with the gene *mcrA*, encoding methyl-coenzyme M reductase, being a prevalent core transcript in methanogenesis. Additionally, an analysis of KEGG modules related to methanogenesis from various sources revealed higher gene expression levels for methanogenesis from acetate and methylamines compared to that from hydrogen and CO_2_ (Fig. 3C; Table S9). Meanwhile, transcripts associated with methanotrophy included K10944 (*pmoA-amoA*), K10945 (*pmoB-amoB*), K10946 (*pmoC-amoC*), and K23995 (*xoxF*), with *pmoC-amoC* being the most abundant. The effect sizes of warming and precipitation alteration were generally small (β = −0.47–0.54; Note SB2) for these methane metabolism transcripts. However, a significant interaction effect of the precipitation level and warming was observed for *pmoB-amoB*, a methane oxidation gene (β = −1.55, *P* = 0.033) (Fig. 3D; Table S10). This also aligned well with the abundance of methanotrophic bacteria, which was increased in warmer and drier soils but declined in warmer and wetter soils (Fig. 2D).

### Shifts of microbial carbon metabolic preferences under climate change

**Fig 4:**
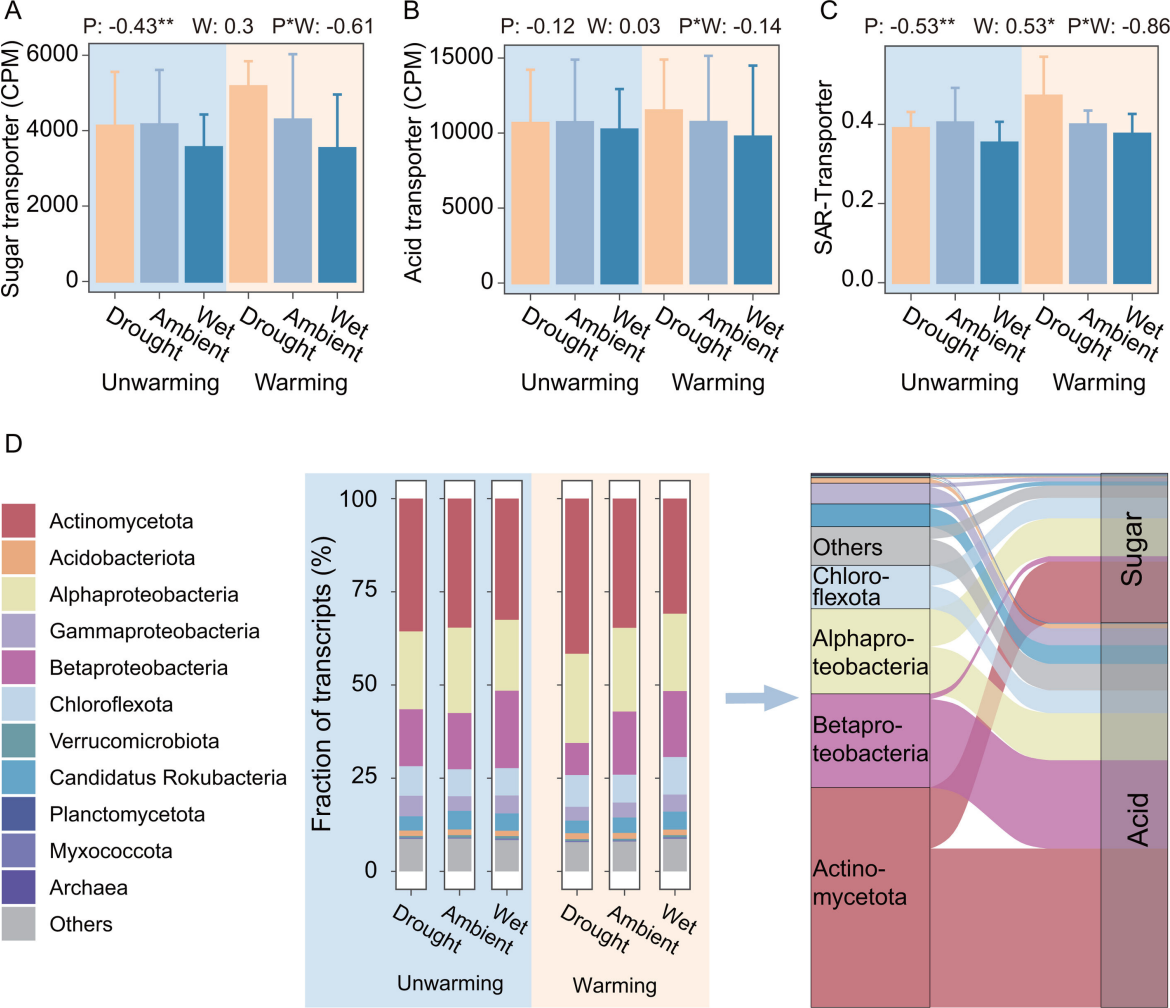
Responses and taxonomical affiliations of sugar and acid transporters. (**A-C**) Responses of the relative abundance of transcripts associated with sugar transporters (**A**) and acid transporters (**B**) and SAR-Transporter (**C**) to experimental treatments. The values at the top of each subplot represented the effect sizes of treatments determined by LMMs [relative abundance/SAR ~ altered precipitation*warming + (1|block)]. Type II Wald χ tests were used to determine statistical significance: **P < 0.01, *P < 0.05. Abbreviations in subplots: P, altered precipitation; W, warming; P*W, interaction between altered precipitation and warming. (**D**) Linking microbial carbon metabolic preferences with active microbes. Proportion of the relative abundance of taxonomical affiliations of transcripts associated with sugar or acid transporters (left). Sankey diagram showing the contributions of active microbes to sugar and acid transporters (right).

Another crucial question is whether and how climate change influences microbial carbon metabolic preferences, especially their preferences for sugars or acids. Emerging evidence posits that microbial substrate preferences can be inferred from genomic content (23). Applying this genomic framework, we analyzed transcripts related to transporters and catabolism of sugars and acids. Overall, the abundance of these transporters and catabolism genes decreased with the precipitation level (β = −0.28, *P* = 0.038 for transporters and β = −0.20, *P* = 0.021 for catabolism genes) (Fig. S3; Table S11). Further, the relative abundance of sugar transporters significantly decreased by 14.58% under wet treatment (β = −0.43, *P* = 0.008 of the precipitation level), while that of acid transporters remained unchanged (Fig. 4A and B). As a result, the sugar-to-acid ratio by transporters (SAR-Transporter) significantly decreased with the precipitation level (β = −0.53, *P* = 0.002) (Fig. 4C; Table S11), which was consistent with our hypothesis. Similarly, the sugar-to-acid ratio by catabolism genes (SAR-Catabolism) also showed a negative correlation with the precipitation level (β = −0.36, *P* = 0.017) (Fig. S4C; Table S11). This could be due to an 11.92% reduction in sugar catabolism (β = −0.25, *P* = 0.009), but only an 8.73% reduction in acid catabolism (β = −0.18, *P* = 0.026) under the wet treatment (Fig. S4A and B). In addition, warming significantly increased the SAR-Transporter (β = 0.53, *P* = 0.048; Fig. 4C), but not the SAR-Catabolism (β = 0.30, *P* = 0.245; Fig. S4C). This pattern mirrors earlier findings from this manipulative experiment, wherein warming elevated soil sugar concentrations in 2015 (Fig. S5) (52).

Taxonomic resolution revealed domain-specific responses: both bacterial and archaeal SAR-Transporter declined in wetter soils (β = −1.05 to −0.52, *P* < 0.10), whereas bacterial SAR-Transporter increased under warming (β = 0.52, *P* = 0.052) (Fig. S6). Methanotrophic bacteria showed no precipitation- or warming-driven SAR-Transporter shifts (*P* > 0.1). Notably, transcripts for sugar/acid transporters in methanotrophic or methanogenic archaea were undetected, potentially reflecting limitations in sequencing depth.

We further explored the main microbial members who actively utilized the substrates of sugars or acids by taxonomically annotating the transcripts of transporters. Actinomycetota and Alphaproteobacteria were important contributors to transcripts involved in both acid and sugar transporters, whereas Betaproteobacteria primarily contributed to transcripts related to acid transporters (Fig. 4D). Notably, the relative abundance of transporters from Betaproteobacteria significantly increased with the precipitation level (β = 0.55, *P* = 0.014), while those from Actinomycetota and Alphaproteobacteria significantly decreased (β = −0.43 to −0.30, *P* < 0.1) (Table S12). This is also consistent with the patterns of the total abundance of Betaproteobacteria (β = 0.87, *P* = 0.015 of the precipitation level), Actinomycetota (β = −0.06, *P* = 0.057), and Alphaproteobacteria (β = −0.07, *P* = 0.046) (Fig. 2C). Regarding archaeal transporter transcripts, we observed that all transcripts from Nitrososphaeria were linked to acid transporters (Fig. S7).

### Linkage of microbial carbon metabolism with soil carbon fluxes

A key follow-up question is how soil microbial metabolic processes are linked to CO_2_ and CH_4_ fluxes. Pairwise correlation analysis revealed that CH_4_ flux was significantly correlated with soil microclimate variables of temperature and pH, as well as microbial gene expressions related to carbohydrate and methane metabolism (*r* = −0.58–0.43, *P* < 0.05) (Fig. S8; Table S13; Note SC). In contrast, CO_2_ flux showed a significant positive correlation only with the relative abundance of transcripts associated with glycosyltransferases (*r* = 0.33, *P* = 0.055). Additionally, the CH_4_/CO_2_ ratio was significantly correlated with all measured soil microclimate variables and most microbial metabolic processes (*r* = −0.61 to 0.47, *P* < 0.1), with the significance primarily driven by CH_4_ (Fig. S8; Note SC). Consistent with patterns in methanogenesis gene expression, the relative abundance of methanogenic archaea was also positively correlated with CH_4_ flux and the CH_4_/CO_2_ ratio (*r* = 0.50–0.55, *P* < 0.01) (Fig. S9).

**Fig 5:**
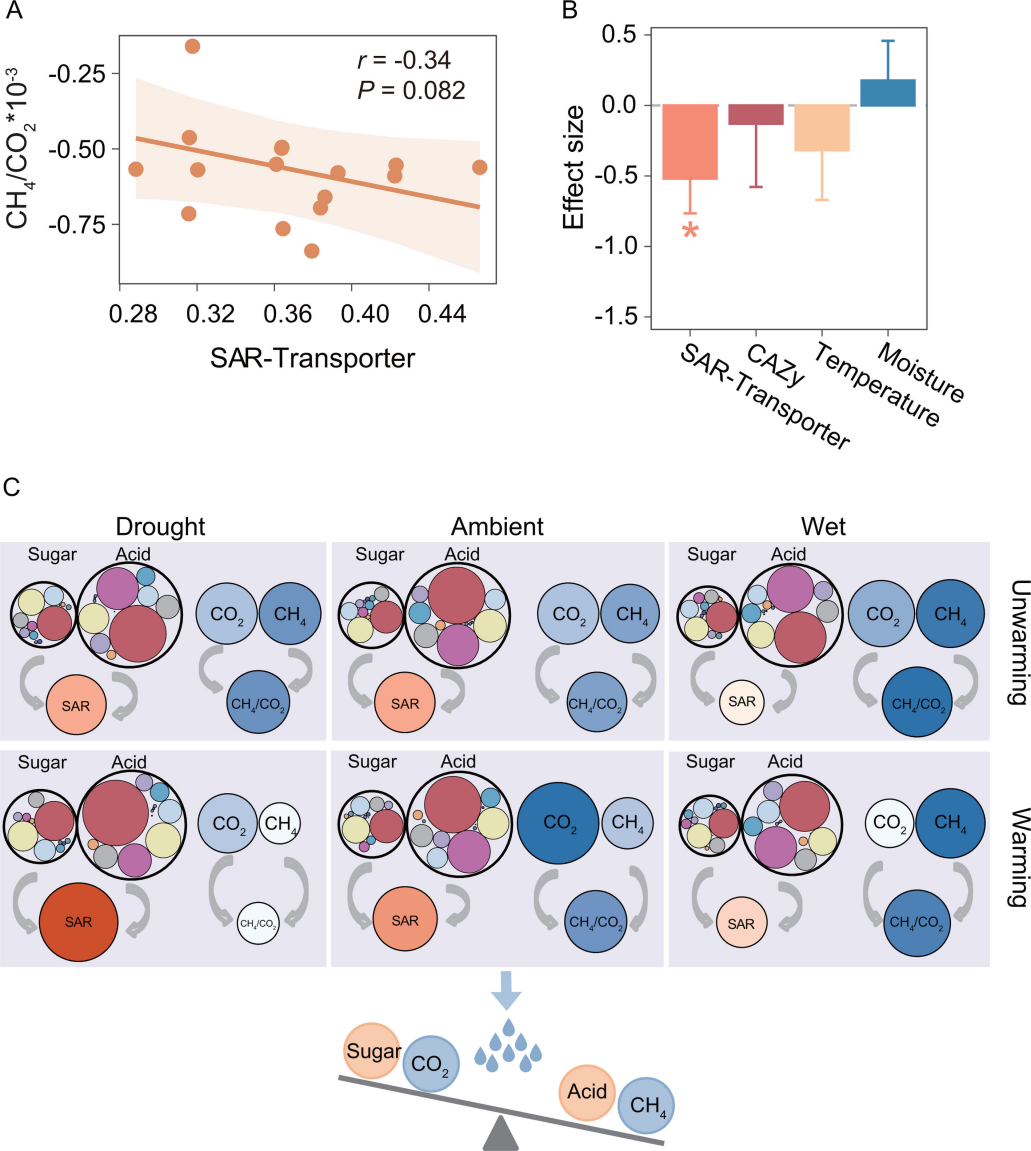
Linking microbial carbon metabolic preferences to CH_4_/CO_2_. (**A**) Correlation between SAR-Transporter and CH_4_/CO_2_. *r* represented the correlation coefficient determined by LMMs [CH_4_/CO_2_ ~SAR-Transporter + (1|block)], and *P* represented the statistical significance of LMMs in the corner of subplot. (**B**) Effects of environmental variables and microbial carbon metabolism on CH_4_/CO_2_. Standardized regression coefficients of LMMs [CH_4_/CO_2_ ~soil moisture + soil temperature + SAR-Transporter + CAZy + (1|block)] represented the effect sizes of environmental variables and microbial carbon metabolism on CH_4_/CO_2_ based on rescaled predictors. Type II Wald χ tests were used to determine the statistical significance: *P < 0.05. (**C**) Linking microbial carbon metabolic preferences with soil carbon fluxes. The size of the circles represented the magnitude of similar indexes. In the sugar and acid transporters, the colors of the circles indicated transcript taxonomy (see Fig. 4). For CO_2_, CH_4_, CH_4_/CO_2_, and SAR, the colors of the circles represented the magnitude of the values, with darker colors indicating higher values. The data excluded transcripts without taxonomic annotation. Sugar, acid, and SAR represented sugar transporters, acid transporters, and SAR-Transporter, respectively. CO_2_ represented the heterotrophic respiration flux.

Importantly, we observed a negative correlation between the CH_4_/CO_2_ ratio and both SAR-Transporter (*r* = −0.34, *P* = 0.082) and SAR-Catabolism (*r* = −0.48, *P* = 0.034) (Fig. 5A; Fig. S4D). We further examined the relationships between the CH_4_/CO_2_ ratio and SAR-Transporter across different domains and functional groups. Similarly, bacterial SAR-Transporter was negatively correlated with the CH_4_/CO_2_ ratio (*r* = −0.35, *P* = 0.076) (Fig. S10). However, SAR-Transporter affiliated with archaea and methanotrophic bacteria showed no significant correlation with the CH_4_/CO_2_ ratio (*P* > 0.1). To account for environmental variables, we conducted a multiple regression analysis, which confirmed that, despite the co-variation of SAR with environmental variables, SAR remained the most influential factor. Standardized regression coefficients showed that SAR-Transporter exerted the largest negative effect on the CH_4_/CO_2_ ratio (β = −0.52, *P* = 0.036), when accounting for carbohydrate metabolism, soil temperature, and moisture (Fig. 5B; Table S14). Although statistically insignificant, SAR-Catabolism also exhibited the largest negative effect on the CH_4_/CO_2_ ratio (β = −0.90, *P* = 0.135) (Fig. S4E). These findings based on microbial community traits suggest that soil microbial communities may favor acids over sugars as carbon substrates in wetter soils, potentially leading to a rise in CH_4_ relative to CO_2_ emissions (Fig. 5C).

## DISCUSSION

Our knowledge of how climate change affects soil microbial carbon metabolism remains limited. This study demonstrates that warming and altered precipitation have significant interactions on microbial carbon metabolism (Fig. 3). Specifically, warming and wet conditions suppressed microbial carbohydrate metabolism and methane oxidation, while warming and drought enhanced these processes. Soil microbial carbon metabolism is influenced by multiple abiotic factors, such as soil temperature, moisture, redox potential, and substrate availability, all of which can be modified by warming and altered precipitation (1, 4, 53). A recent study reveals that soil moisture was positively correlated with gene abundance of microbial carbon metabolism, such as glycoside hydrolases (54). Increased precipitation may result in a reduction in dissolved organic carbon (31) and an increase in recalcitrant carbon (55), leading to increased microbial investment in glycoside hydrolases. Additionally, increased precipitation elevates soil moisture, which influences redox potential by altering oxygen availability in the soil, thus promoting methanogenesis (53, 56, 57). Conversely, warming may reduce soil moisture by enhancing evapotranspiration (58) and affect dissolved organic carbon (31, 59), potentially offsetting the effects of increased precipitation on microbial carbon metabolism. These findings underscore the significant interaction between warming and precipitation alterations, highlighting the importance of long-term, multifactorial field experiments to accurately predict future ecosystem responses under complex climate change scenarios.

It is widely accepted that reduced precipitation and soil drying enhance oxygen availability, thereby promoting methane oxidation (56, 60). However, our observations indicate that methanotrophic gene expressions increased with higher precipitation under ambient temperature conditions but decreased with increased precipitation under warming conditions (Fig. 2D and 3D). This complex response to changes in warming and precipitation suggests that CH_4_ oxidation is regulated by multiple interacting factors, including methane concentration, redox potential, and temperature (29, 61, 62). Increased precipitation can elevate CH_4_ concentrations, further stimulating methane oxidation (61, 63), as CH_4_ production and oxidation occur simultaneously (64). In wetter soil, oxygen limitation is generally considered a constraint on methane oxidation (65). However, the presence of alternative-to-oxygen electron acceptors may alleviate oxygen availability limitation and promote methane oxidation (66, 67). Warming may reduce methane oxidation by suppressing methanogenesis due to decreased soil water content (61). The response of CH_4_ metabolism to climate change is complex and depends on soil physicochemical properties.

The response of microbial metabolic preferences for different carbon substrates to climate change remains poorly understood. A key finding of this study is that under increased precipitation, soil microbial communities exhibited a shift toward acid metabolism over sugar metabolism, as evidenced by the differential expression of transcripts associated with sugar and acid transporters, as well as catabolic enzymes (Fig. 4; Fig. S4). This observation aligns with previous studies; for example, a study conducted in a semi-arid grassland demonstrated that increased precipitation may primarily enhance bacterial utilization of amino acids, while warming mainly increases carbohydrate utilization (68). Similarly, metagenomic sequencing revealed that water addition could drive soil microbes to invest more resources in amino acid degradation and less in simple carbohydrate utilization (32). The primary microbial taxa contributing to the shift towards acid metabolism was Betaproteobacteria, whose transcripts enriched under the elevated precipitation (Fig. 2C and 4D). Some taxa of Betaproteobacteria, such as *Polynucleobacter necessarius,* which lack the glycolytic pathway and are unable to utilize sugars as carbon (69), have a strong preference for acid carbon substrates, such as acetate and succinate (70). This preference for acid metabolism was also observed in archaeal communities in wetter soils (Fig. S6). Archaea, such as Nitrososphaeria, produce diverse organic acids through the hydroxypropionate/hydroxybutyrate cycle for biosynthesis (71, 72). Furthermore, the community-level shift toward acid metabolism may result from changes in the availability of soil carbon substrates under climate change (52).

Our results indicate a negative relationship between microbial sugar-to-acid metabolic preferences and soil CH_4_/CO_2_ emission ratio, even after accounting for variations in soil microclimate conditions and microbial carbohydrate metabolism (Fig. 5). Notably, variation in the CH_4_/CO_2_ ratio was primarily driven by changes in CH_4_ flux rather than CO_2_ (Fig. S8). This pattern may be attributed to the fact that altered precipitation primarily influenced CH_4_ emissions, while experimental warming had no significant effect (Fig. 1). These findings align with prior studies suggesting that long-term warming can lead to acclimatization of soil respiration through shifts in microbial community composition and reductions in carbon decomposition (6, 73).

The observed negative association between microbial sugar-to-acid metabolic preferences and the soil CH_4_/CO_2_ ratio may be explained by the role of acid metabolism in supporting methanogenesis. Organic acids, such as lactate, butyrate, and propionate, can be degraded into methanogenic substrates by various specialized microbes working collectively (74, 75). For example, studies on anaerobic digestion have shown that diverse hydrolytic and acidogenic bacteria can rapidly degrade carbon substrates, thereby enhancing the CH_4_ emission by providing methanogens with abundant utilizable substrates, such as H_2_/CO_2_ and acetate (76). Furthermore, meta-analysis suggests that the effects of altered precipitation on soil CH_4_ flux depend on soil moisture (77). Wet conditions can reduce oxygen availability, promoting anaerobic metabolism, which favors the accumulation of fermentation products (e.g., lactate, propionate, and acetate) that methanogens can utilize (28, 76). Consistent with this, our results show that sugar-to-acid metabolic preferences were negatively correlated with methanogenesis gene expression, but not with methanotrophy-related genes (Fig. S8).

In addition to supporting methanogenic activity, acid metabolism may also contribute to the biosynthesis of methanogens. For instance, the recently isolated methyl-reducing methanogen *Candidatus* Methanosuratincolia lacks sugar transporters specific for the glycolysis pathway and utilizes acetate for gluconeogenesis, a significant biosynthetic pathway in methanogens (78).

However, we did not observe increased transcript abundance of acetoclastic methanogenesis in wetter soils (Fig. 3C), nor did we detect transcripts encoding sugar or acid transporters affiliated with methanogenic or methanotrophic archaea. These patterns may reflect limitations in sequencing depth, which likely reduced the detection of archaeal transcripts. Future studies should increase sequencing depth to enable a more comprehensive analysis of archaeal carbon metabolic preferences, particularly those of methanogens.

Members of the Betaproteobacteria, which dominate the acid metabolic preference in wetter soils, may influence CH_4_ cycling through impacts on redox conditions and substrate availability (28, 79, 80). Specifically, many Betaproteobacteria encode enzymes involved in denitrification, thereby depleting energetically favorable electron acceptors such as NO_3_^-^ and creating more favorable conditions for methanogenesis (28, 79). Betaproteobacteria may also contribute to methanogenesis by providing substrates through gluconeogenesis (69). Thus, a community-level shift from sugar to acid metabolism—primarily driven by Betaproteobacteria—may indirectly promote methanogenesis.

Collectively, our findings suggest that substrates and microbial biomass generated via acid catabolism may enhance methanogenesis, potentially leading to an increased CH_4_/CO_2_ emission ratio in wetter soils. We then propose the potential use of the sugar-to-acid ratio as a community-level trait for predicting shifts in CH_4_ emissions relative to CO_2_. By monitoring changes in microbial carbon substrate metabolic preferences, this trait could serve as an early indicator of increasing CH_4_ emissions in response to environmental stressors.

In summary, we assessed *in situ* soil microbial activity through the long-term experiments of climate manipulation in a Tibetan alpine grassland, which provides critical insights into how climate warming and altered precipitation modulate microbial carbon metabolism and greenhouse gas emissions. Our findings reveal a significant interaction between warming and precipitation alterations, where warming and wet conditions generally suppressed microbial carbohydrate metabolism and methane oxidation, while warming and drought enhanced these processes. The interaction between warming and precipitation needs further exploration to refine predictions of carbon cycling under future climate scenarios. Notably, current research emphasizes the role of alpine grasslands as a net CH_4_ sink, mitigating global warming (31, 81, 82). However, our findings suggest that increased precipitation shifted microbial communities towards acid metabolism over sugar metabolism, a transition primarily driven by taxa such as Betaproteobacteria. This metabolic shift is associated with an increased CH_4_/CO_2_ emission ratio, suggesting that microbial substrate metabolic preferences play a pivotal role in regulating carbon-climate feedbacks. However, our study is based on a single soil sampling, which may limit the generalizability of the observed microbial dynamics. Future studies should incorporate long-term time series and multisite sampling to investigate how shifts in microbial substrate metabolic preferences across various ecosystems influence carbon fluxes. Incorporating this community-level trait into carbon cycling models will enable more accurate assessments of global carbon-climate feedbacks.

## Data Availability

The metatranscriptomes are deposited in the National Center for Biotechnology Information (NCBI) under project accession number PRJNA1171591. Data that further support the findings of this study are available at https://doi.org/10.6084/m9.figshare.29314286.v1. R scripts for statistical analyses are available on GitHub at https://github.com/XiaominF/Haibei-metatranscriptomics.

## SUPPLEMENTAL MATERIAL

Supplemental information msystems.00470-25-s0001.docx Notes SA to SC, Figures S1 to S10, and Tables S1 to S14.

ASM does not own the copyrights to Supplemental Material that may be linked to, or accessed through, an article. The authors have granted ASM a non-exclusive, world-wide license to publish the Supplemental Material files. Please contact the corresponding author directly for reuse.
