# Supplementary material for: Long‐term elevated precipitation promotes an acid metabolic preference in soil microbial communities in a Tibetan alpine grassland
Source: mSystems. 2025 Jul 28;10(8):e00470-25. doi: 10.1128/msystems.00470-25 (PMC12363203; doi:10.1128/msystems.00470-25)
Supplement: Supplemental information — Notes SA to SC, Figures S1 to S10, and Tables S1 to S14. [file msystems.00470-25-s0001.docx]

# Supplementary information for

**Long‐term elevated precipitation promotes an acid metabolic preference in soil microbial communities in a Tibetan alpine grassland**

Xiaomin Fan, Xue Guo, Qi Qi, Haoran Gui, Yujiang Li, Yunfeng Yang, Jin-Sheng He, Linwei Wu

Correspondence to: linwei.wu@pku.edu.cn, and jshe@pku.edu.cn

**This file includes:**

Supplementary Notes A-C

Supplementary Figures 1 to 10

Supplementary Tables 1 to 14

**Supplementary Notes**

**A.** **Treatment effects on active microbial members**

We quantified the effects of warming and altered precipitation and their interactions on active microbial members by LMMs. The relative abundance of Actinomycetota (*β* = -0.06, *P* = 0.057) and Alphaproteobacteria (*β* = -0.07, *P* = 0.046) decreased with elevated precipitation (Fig. 2C and Table S5). While altered precipitation had no significant effect on the relative abundance of Chloroflexota (*β* = 0.17, *P* = 0.226). However, we found the significant negative interaction effects of the precipitation level and warming on the relative abundance of both Actinomycetota (*β* = -1.27, *P* = 0.068), Alphaproteobacteria (*β* = -1.35, *P* = 0.057) and Chloroflexota (*β* = -1.16, *P* = 0.064).

The different lineages of methanotrophs and methanogens may respond differentially to the treatments. For the methanotrophic bacteria, altered precipitation had positive effects on Methylococcus and Methylomicrobium (*β* = 0.47 - 0.75, *P* < 0.05), and negative effect on Methylocystis (*β* = -0.19, *P* = 0.063), independent of warming (Table S7). However, the precipitation level and warming had significant or marginally significant interaction effects on Methylocaldum, Methylohalobius, Methylosarcina, and Methylosinus (*β* = -1.89 - 1.26, *P* < 0.1) (Fig. S2 and Table S7).

**B. Treatment effects on microbial carbohydrate and methane metabolism**

**1. Carbohydrate metabolism**

LMMs were further used to test the effects of warming and altered precipitation and their interactions on microbial carbohydrate metabolism. We found that warming and altered precipitation had a significant negative interaction on the relative abundance of transcripts involved in glycoside hydrolases (*β* = -1.75, *P* = 0.018) (Fig. 3A and Table S8), that is, glycoside hydrolases abundance increased (19.73%) in warmer and drier soils, but declined (1.46%) in warmer and wetter soils compared to ambient temperature and precipitation. For the glycosyltransferases, the analysis indicated that the precipitation level had a negative effect on the relative abundance of transcripts involved in glycosyltransferases (*β* = -0.07, *P* = 0.014), while warming had a positive impact on it (*β* = 0.58, *P* = 0.049). Moreover, we found a significant negative interaction of the precipitation level and warming on the relative abundance of transcripts involved in glycosyltransferases (*β* = -1.57, *P* = 0.018), showing that glycosyltransferases abundance increased 20% in warmer and drier soils, and declined 5.12% in warmer and wetter soils.

**2. Methane metabolism**

The results of methane metabolism indicated that altered precipitation and warming had no significant effects on gene expression associated with hydrogenotrophic methanogenesis, such as *mch*, *ftr*, *mer*, *fwdC*, *fwdB*, *fwdA* (warming: *β* = -0.06 - 0.44, *P* > 0.1, precipitation level: *β* = -0.22 - 0.01, *P* > 0.1) (Fig. 3C and Table S10). For the aceticlastic methanogenesis, we found that the relative abundance of transcripts associated with *ACSS1*_*2* negatively correlated with the precipitation level (*β* = -0.04, *P* = 0.052), while positively correlated with warming (*β* = 0.50, *P* = 0.098). Specially, warming and altered precipitation had a significant negative interaction effect on it (*β* = -1.31, *P* = 0.056). About the methylotrophic methanogenesis and core methanogenesis, the analysis indicated that altered precipitation negatively affected the relative abundance of transcripts involved in *mcrC* and *mttB* (*β* = -0.23 - -0.22, *P* < 0.1). Among the methanotrophy, the expressions of *pmoA-amoA*, *pmoC-amoC* and *xoxF* were not affected by warming and altered precipitation (warming: *β* = -0.17 - 0.07, *P* > 0.1, precipitation level: *β* = -0.33 - 0.06, *P* > 0.1).

**C. Correlations between microbial carbon metabolism and soil carbon fluxes**

The pairwise correlation analysis indicated that CH_4_ flux was negatively correlated with soil temperature (r = -0.56, *P* = 0.026) and pH (r = -0.58, *P* = 0.001) (Fig. S8 and Table S13). Similarly, we found the negative correlations between CH_4_ flux and the relative abundance of transcripts associated with glycosyltransferases (r = -0.47, *P* = 0.024) and polysaccharide lyases (r = -0.46, *P* = 0.048). In contrast, CH_4_ flux increased with the rise in the relative abundance of transcripts involved in methanogenesis (r = 0.43, *P* = 0.043). About CH_4_/CO_2_, soil temperature (r = -0.43, *P* = 0.026) and pH (r = -0.47, *P* = 0.011) had the negative effects on CH_4_/CO_2_, while soil moisture had the positive effect (r = 0.47, *P* = 0.064). CH_4_/CO_2_ was negatively correlated with the gene expresses involved in carbohydrate metabolism, such as carbohydrate binding, carbohydrate esterases, glycosyltransferases, polysaccharide lyases (r = -0.61 - -0.40, *P* < 0.1). Moreover, the relative abundance of transcripts involved in sugar transporter had the negative effect on CH_4_/CO_2_ (r = -0.43, *P* = 0.064), while methanogenesis had a positive effect on CH_4_/CO_2_ (r = 0.38, *P* = 0.064) (Fig. S8 and Table S13).

**Supplementary Figures**


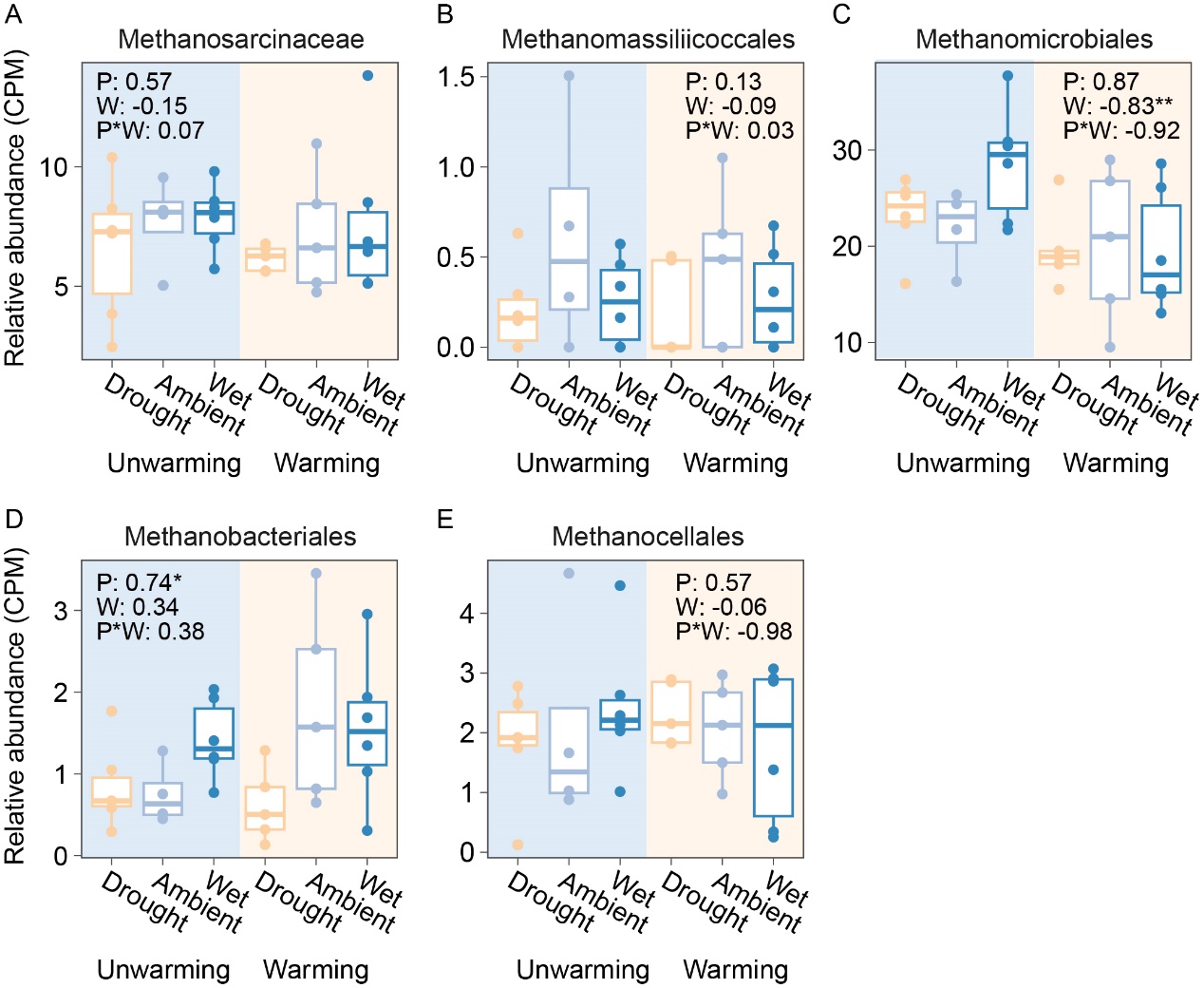


**Fig. S1 Relative abundance of taxonomic affiliations of transcripts associated with methanogenic archaea at the order-level.** The values in the corner of each subplot represented the effect sizes of treatments on them (LMMs: relative abundance ~ altered precipitation*warming + (1|block)). Type II Wald chi-square tests were used to determine statistical significance: ** *P* < 0.01, * *P* < 0.05. Abbreviations in subplots: P, altered precipitation; W, warming; P*W, interaction between altered precipitation and warming. The Methanosarcinales order, which includes anaerobic methanotrophs, utilized only transcripts affiliated with the Methanosarcinaceae family.

**
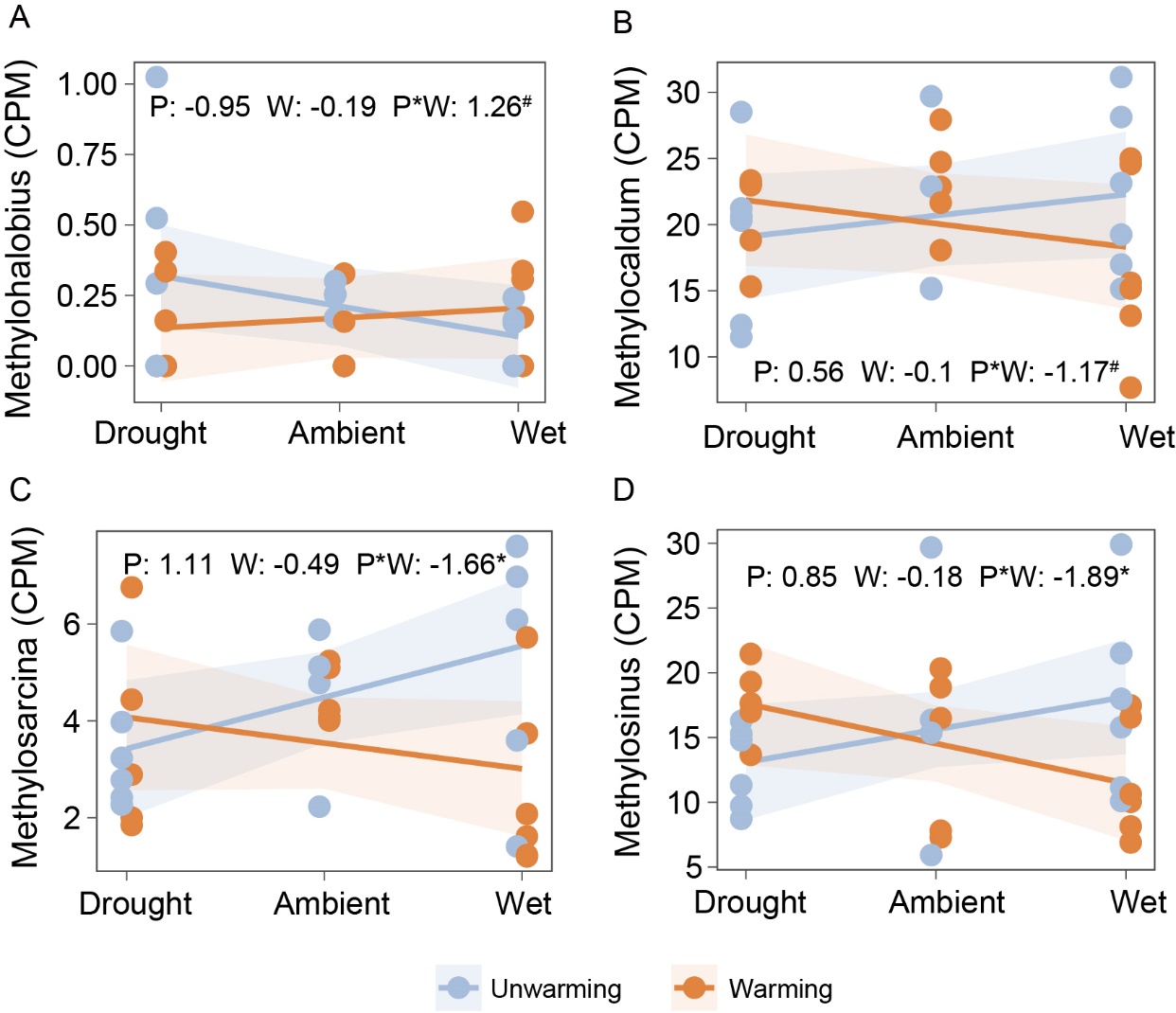
**

**Fig. S2 Relative abundance of taxonomic affiliations of transcripts associated with methanotrophic bacteria.** The values in the corner of each subplot represented the effect sizes of treatments on them (LMMs: relative abundance ~ altered precipitation*warming + (1|block)). Type II Wald chi-square tests were used to determine statistical significance: * *P* < 0.05, ^#^ *P* < 0.1. Abbreviations in subplots: P, altered precipitation; W, warming; P*W, interaction between altered precipitation and warming.

**
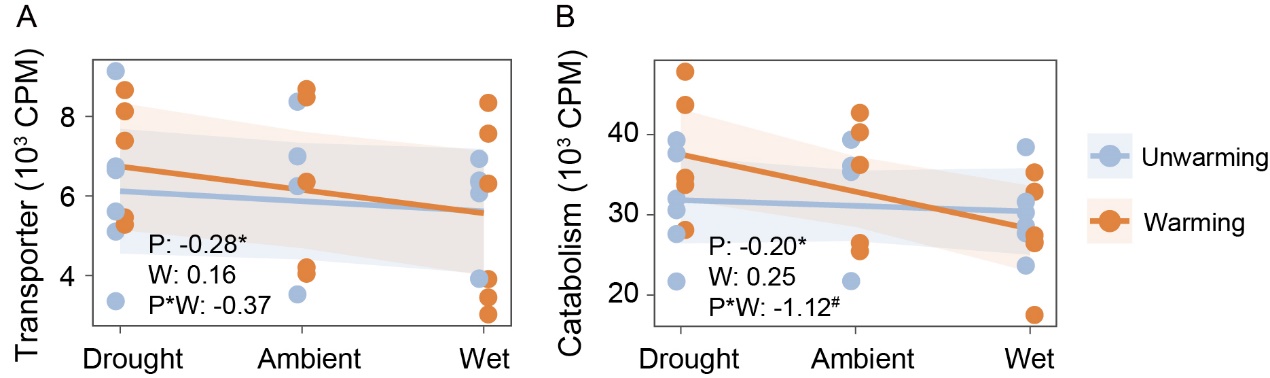
**

**Fig. S3 Relative abundance of transcripts related to transporters (A) and catabolism (B) of sugars and acids.** The values in the corner of each subplot represented the effect sizes of treatments on them (LMMs: relative abundance ~ altered precipitation*warming + (1|block)). Type II Wald chi-square tests were used to determine statistical significance: * *P* < 0.05, ^#^ *P* < 0.1. Abbreviations in subplots: P, altered precipitation; W, warming; P*W, interaction between altered precipitation and warming.


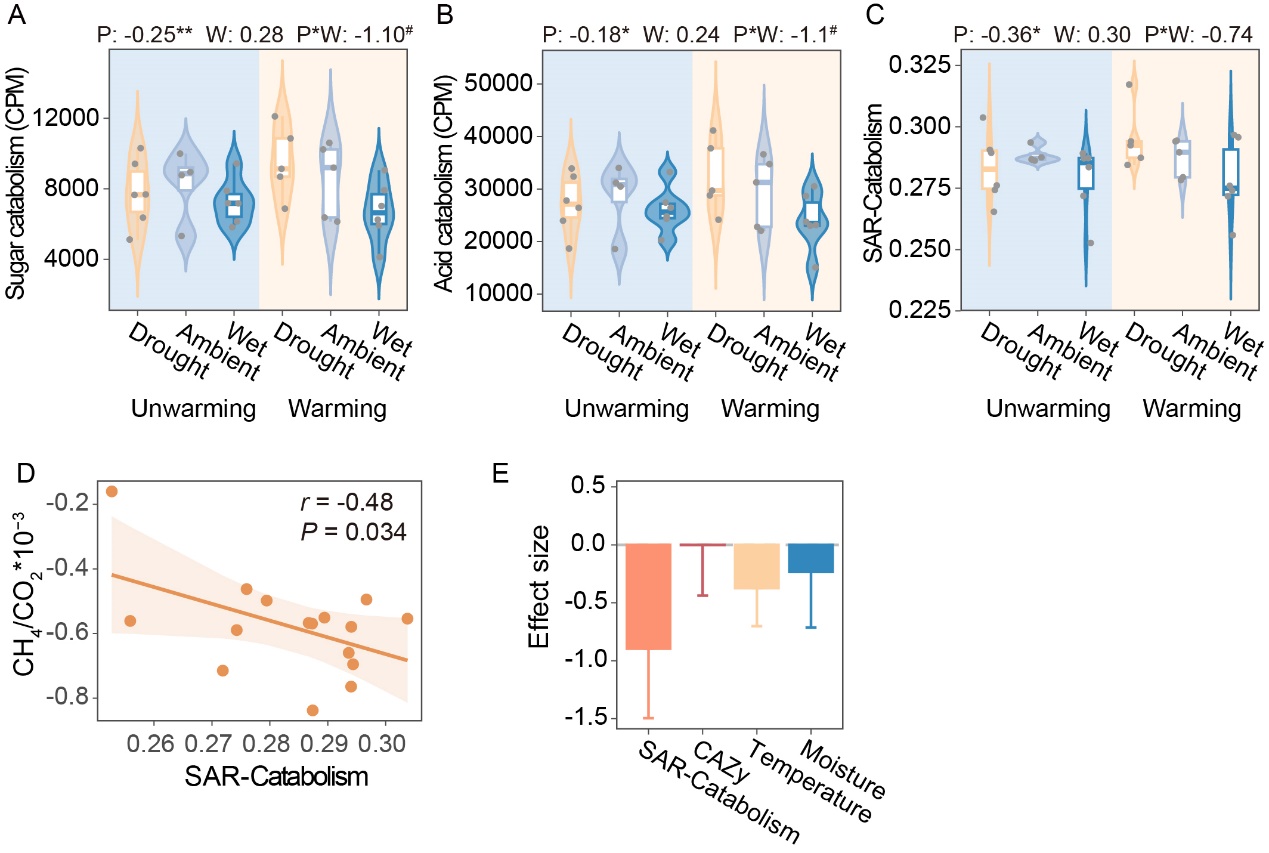


**Fig. S4 Relative abundance of transcripts associated with sugar and acid catabolism and their correlations with CH_4_/CO_2_.** A-C, Responds of the relative abundance of transcripts associated with sugar catabolism (A) and acid catabolism (B), and SAR-Catabolism (C) to experimental treatments. The values at the top of each subplot represented the effect sizes of treatments determined by LMMs (relative abundance/SAR ~ altered precipitation*warming + (1|block)). Type II Wald chi-square tests were used to determine statistical significance: ** *P* < 0.01, * *P* < 0.05, ^#^ *P* < 0.1. Abbreviations in subplots: P, altered precipitation; W, warming; P*W, interaction between altered precipitation and warming. D, Correlation between CH_4_/CO_2_ and SAR-Catabolism. The correlation coefficient (*r*) determined by LMMs (CH_4_/CO_2_ ~ SAR + (1|block)). E, Effects of environmental variables and microbial carbon metabolism on CH_4_/CO_2_. Standardized regression coefficients of LMMs (CH_4_/CO_2_ ~ soil moisture + soil temperature + SAR-Catabolism + CAZy + (1|block)) represented the effect sizes of environmental variables and microbial carbon metabolism on CH_4_/CO_2_ based on rescaled predictors.


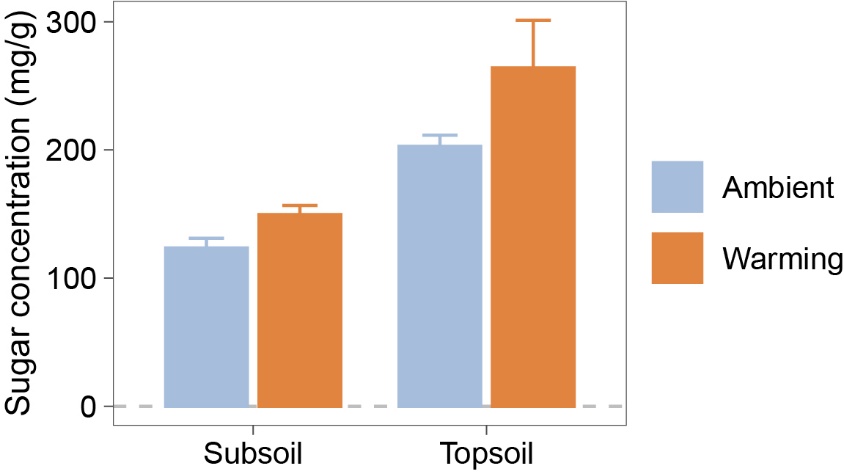


**Fig. S5 Sugar concentrations in top- and subsoils under ambient and warming treatments.** Data were mean ± standard error (refer to raw concentration data in Table S2 (1)).


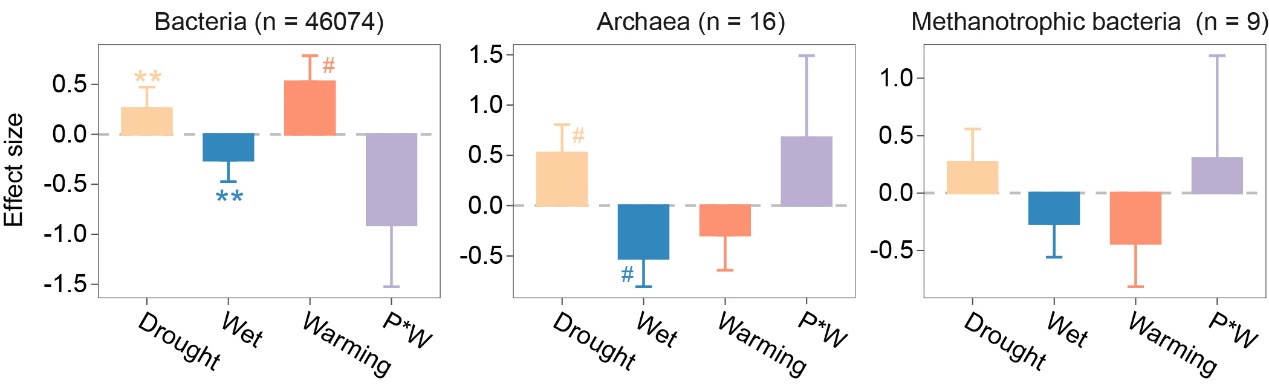


**Fig. S6 Effects of experimental treatments on SAR-Transporter of bacteria, archaea, and methanotrophic bacteria.** The n value in parentheses represented the corresponding number of transcripts. Data were coefficients and standard errors of LMMs. Type II Wald chi-square tests were used to determine statistical significance: ** *P* < 0.01, ^#^ *P* < 0.1.


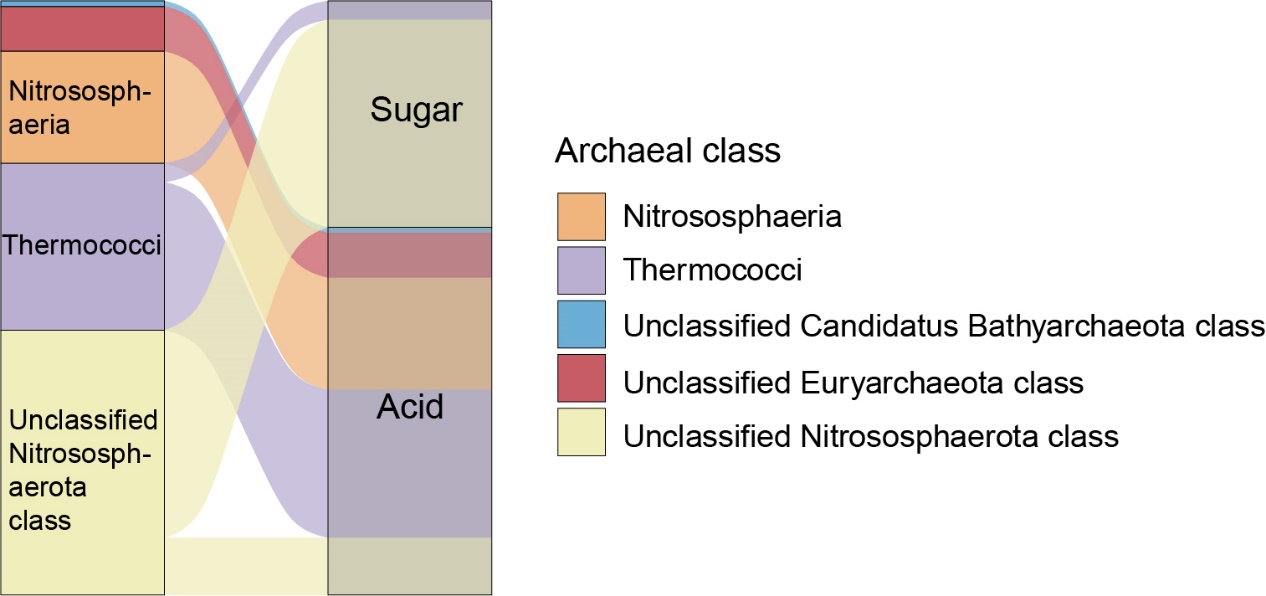


**Fig. S7 Contributions of active archaea to sugar and acid transporters.**


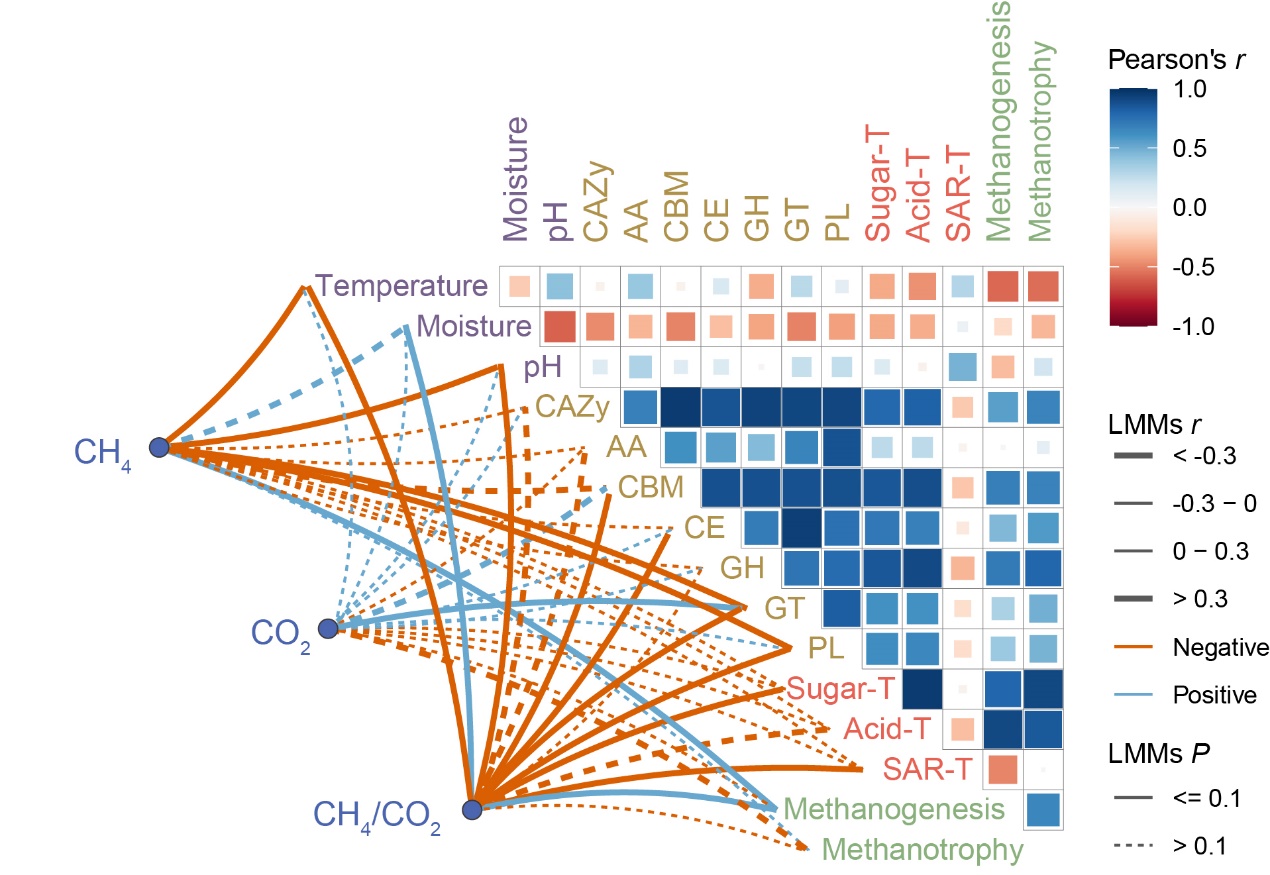


**Fig. S8 Correlations between soil carbon fluxes and environmental variables and microbial carbon metabolism.** Edge width equaled to the absolute value of the correlation coefficient determined by the LMMs (flux/ratio ~ environmental variable/microbial carbon metabolism + (1|block)). Colors of the lines indicated correlation types. Solid and dashed lines represented the statistical significance determined by Type II Wald chi-square tests. Pairwise comparisons of environmental variables and microbial carbon metabolism were shown in the triangle, utilizing a color gradient denoting Pearson’s correlation coefficient. CO_2_ represented the heterotrophic respiration flux. Methanogenesis included the core methanogenesis, methylotrophic methanogenesis, aceticlastic methanogenesis, and hydrogenotrophic methanogenesis. Sugar-T, Acid-T, and SAR-T represented sugar transporters, acid transporters, and SAR-Transporter, respectively. Carbohydrate metabolism included glycoside hydrolases (GH), glycosyltransferases (GT), polysaccharide lyases (PL), auxiliary activities (AA), carbohydrate esterases (CE), carbohydrate binding (CBM).


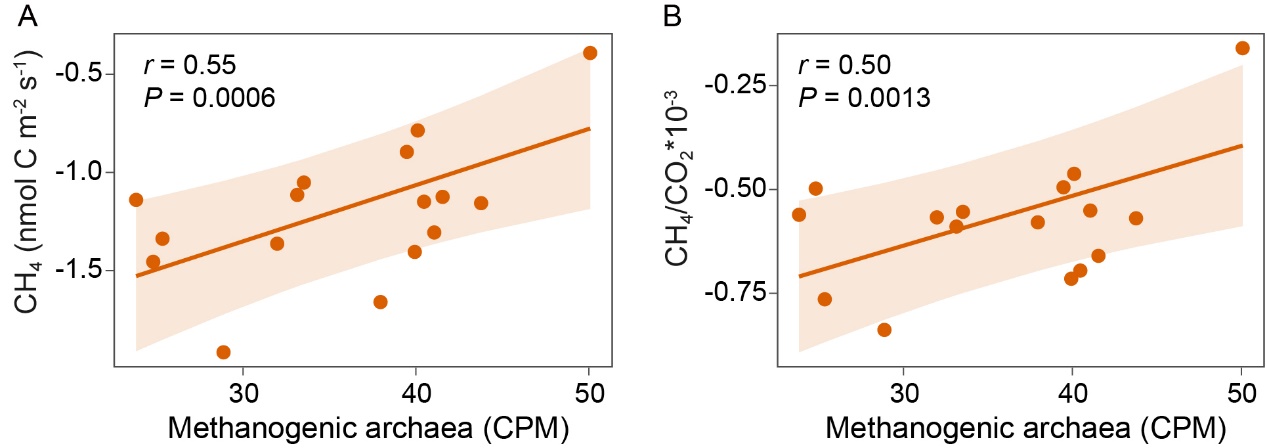


**Fig. S9 Correlations between methanogenic archaea and CH_4_ (A) and CH_4_/CO_2_ (B).** *r* represented the correlation coefficients determined by LMMs (flux/ratio ~ methanogenic archaea + (1|block)), and *P* represented the statistical significance of LMMs determined by Type II Wald chi-square tests in the top left of subplots.


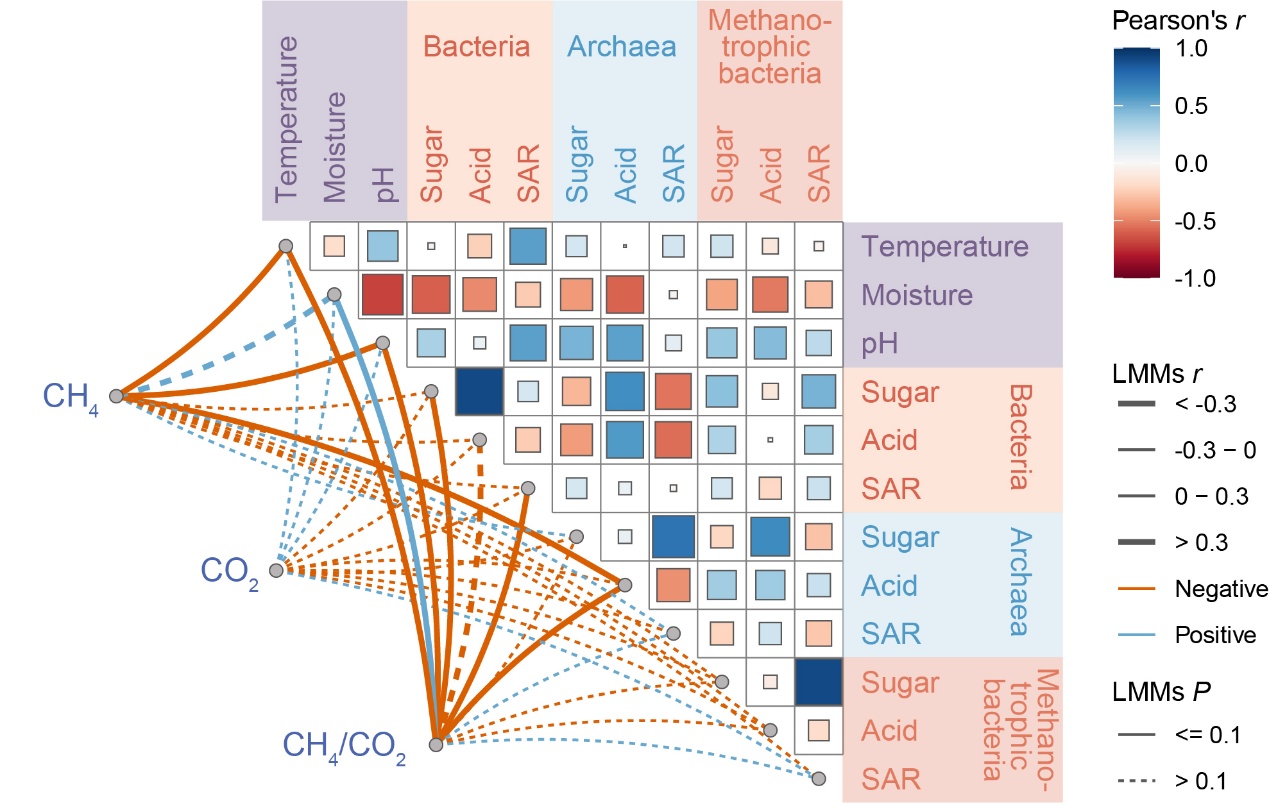


**Fig. S10 Correlations between soil carbon fluxes and environmental variables and microbial carbon metabolic preferences.** Edge width equaled to the absolute value of the correlation coefficient determined by the LMMs (flux/ratio ~ environmental variable/microbial carbon metabolic preferences + (1|block)). Colors of the lines indicated correlation types. Solid and dashed lines represented the statistical significance determined by Type II Wald chi-square tests. Pairwise comparisons of environmental variables and microbial carbon metabolic preferences were shown in the triangle, utilizing a color gradient denoting Pearson’s correlation coefficient. CO_2_ represented the heterotrophic respiration flux. Sugar, Acid, and SAR represented sugar transporters, acid transporters, and SAR-Transporter, respectively.

**Supplementary Tables**

**Table S1. Effects of** **experimental treatments on soil variables and carbon fluxes.** Environmental factors: soil temperature, soil moisture, pH. Microbial biomass carbon: MBC. Ecosystem carbon fluxes: NEE (net ecosystem carbon exchange), ER (ecosystem respiration). Soil carbon fluxes: CH_4_, Rs (soil respiration), Rh (heterotrophic respiration). P, W, and P*W represented altered precipitation, warming, and the interaction of altered precipitation and warming, respectively. Coefficients of LMMs (y ~ altered precipitation*warming + (1|block)) represented the treatment effect sizes. Type II Wald chi-square tests were used to determine the statistical significance. Significant effects (*P* < 0.1) were bolded. R^2^_m_ and R^2^_c_ represented the marginal and conditional coefficients of determination for LMMs, respectively.

| Factor | Treatment | Coefficient | Standard error | t | Chi-square | df | *P* | R^2^_m_ | R^2^_c_ |
| --- | --- | --- | --- | --- | --- | --- | --- | --- | --- |
| **Temperature** | **P** | **-0.71** | **0.47** | **-1.51** | **6.25** | **1** | **0.012** | 0.46 | 0.76 |
| **Temperature** | **W** | **1.37** | **0.27** | **5.04** | **25.44** | **1** | **4.58E-07** |  |  |
| Temperature | P*W | -0.24 | 0.66 | -0.36 | 0.13 | 1 | 0.716 |  |  |
| **Moisture** | **P** | **0.09** | **0.03** | **3.23** | **22.87** | **1** | **1.73E-06** | 0.64 | 0.64 |
| **Moisture** | **W** | **-0.04** | **0.02** | **-2.61** | **6.80** | **1** | **0.009** |  |  |
| Moisture | P*W | 0.01 | 0.04 | 0.22 | 0.05 | 1 | 0.829 |  |  |
| **pH** | **P** | **-0.45** | **0.12** | **-3.63** | **33.00** | **1** | **9.21E-09** | 0.49 | 0.49 |
| pH | W | -0.01 | 0.07 | -0.18 | 0.03 | 1 | 0.859 |  |  |
| pH | P*W | -0.11 | 0.18 | -0.61 | 0.38 | 1 | 0.540 |  |  |
| **MBC** | **P** | **536.03** | **171.28** | **3.13** | **26.99** | **1** | **2.04E-07** | 0.47 | 0.47 |
| **MBC** | **W** | **169.88** | **98.89** | **1.72** | **2.95** | **1** | **0.086** |  |  |
| MBC | P*W | 186.44 | 242.23 | 0.77 | 0.59 | 1 | 0.441 |  |  |
| **NEE** | **P** | **-2.16** | **1.32** | **-1.63** | **15.87** | **1** | **6.79E-05** | 0.34 | 0.59 |
| NEE | W | -0.32 | 0.76 | -0.41 | 0.17 | 1 | 0.678 |  |  |
| **NEE** | **P*W** | **-3.13** | **1.87** | **-1.67** | **2.80** | **1** | **0.095** |  |  |
| ER | P | 0.80 | 0.71 | 1.12 | 2.27 | 1 | 0.132 | 0.06 | 0.36 |
| ER | W | -0.03 | 0.41 | -0.07 | 0.00 | 1 | 0.948 |  |  |
| ER | P*W | -0.07 | 1.01 | -0.07 | 0.01 | 1 | 0.941 |  |  |
| **CH_4_** | **P** | **0.17** | **0.22** | **0.78** | **5.24** | **1** | **0.022** | 0.26 | 0.44 |
| CH_4_ | W | -0.14 | 0.13 | -1.13 | 1.27 | 1 | 0.260 |  |  |
| CH_4_ | P*W | 0.37 | 0.31 | 1.18 | 1.39 | 1 | 0.238 |  |  |
| Rs | P | -0.04 | 0.23 | -0.19 | 1.11 | 1 | 0.292 | 0.14 | 0.14 |
| Rs | W | 0.00 | 0.13 | 0.00 | 0.00 | 1 | 0.998 |  |  |
| Rs | P*W | 0.44 | 0.33 | 1.33 | 1.76 | 1 | 0.184 |  |  |
| Rh | P | 0.09 | 0.30 | 0.31 | 0.01 | 1 | 0.924 | 0.01 | 0.32 |
| Rh | W | -0.05 | 0.17 | -0.26 | 0.07 | 1 | 0.794 |  |  |
| Rh | P*W | -0.23 | 0.43 | -0.54 | 0.29 | 1 | 0.590 |  |  |
| **CH_4_/CO_2_** | **P** | **0.08** | **0.10** | **0.87** | **5.50** | **1** | **0.019** | 0.28 | 0.43 |
| CH_4_/CO_2_ | W | -0.07 | 0.06 | -1.29 | 1.67 | 1 | 0.197 |  |  |
| CH_4_/CO_2_ | P*W | 0.15 | 0.14 | 1.12 | 1.25 | 1 | 0.263 |  |  |

**Table S2. Proportion of the relative abundance of super-kingdom affiliations of transcripts across all, drought, ambient, wet, drought and warming, warming, as well as wet and warming plots.**

| Super-kingdom | All | Drought | Ambient | Wet | Drought + Warming | Warming | Wet + Warming |
| --- | --- | --- | --- | --- | --- | --- | --- |
| Bacteria | 94.14 | 93.35 | 94.56 | 94.64 | 94.00 | 94.97 | 93.54 |
| Viruses | 3.87 | 4.42 | 3.81 | 3.81 | 3.13 | 3.45 | 4.43 |
| Eukaryota | 1.20 | 1.39 | 0.79 | 0.77 | 2.03 | 0.84 | 1.31 |
| Archaea | 0.28 | 0.29 | 0.31 | 0.28 | 0.32 | 0.25 | 0.24 |
| Others | 0.51 | 0.56 | 0.53 | 0.51 | 0.51 | 0.49 | 0.48 |

**Table S3.** **Proportion of the relative abundance of phylum- or class-level affiliations of bacterial transcripts across all, drought, ambient, wet, drought and warming, warming, as well as wet and warming plots.**

| Phylum/Class | All | Drought | Ambient | Wet | Drought + Warming | Warming | Wet + Warming |
| --- | --- | --- | --- | --- | --- | --- | --- |
| Actinomycetota | 22.73 | 21.47 | 23.49 | 19.82 | 28.34 | 24.41 | 20.09 |
| Acidobacteriota | 17.76 | 18.33 | 17.05 | 19.13 | 16.58 | 15.60 | 19.14 |
| Alphaproteobacteria | 13.43 | 13.11 | 14.30 | 12.15 | 15.37 | 14.20 | 12.12 |
| Gammaproteobacteria | 8.09 | 9.46 | 7.48 | 8.08 | 7.67 | 7.85 | 7.73 |
| Betaproteobacteria | 7.81 | 7.52 | 7.77 | 9.08 | 5.00 | 9.05 | 8.22 |
| Chloroflexota | 3.78 | 3.61 | 3.73 | 3.52 | 4.26 | 3.85 | 3.78 |
| Verrucomicrobiota | 3.03 | 2.97 | 2.92 | 3.29 | 2.32 | 2.93 | 3.63 |
| Candidatus Rokubacteria | 3.02 | 2.90 | 3.33 | 3.26 | 2.63 | 2.88 | 3.16 |
| Planctomycetota | 2.93 | 2.90 | 2.96 | 3.08 | 2.40 | 2.85 | 3.34 |
| Others | 17.41 | 17.73 | 16.96 | 18.59 | 15.43 | 16.38 | 18.80 |

**Table S4. Proportion of the relative abundance of** **class-level affiliations of archaeal transcripts across all, drought, ambient, wet, drought and warming, warming, as well as wet and warming plots.**

| Class | Phylum | All | Drought | Ambient | Wet | Drought + Warming | Warming | Wet + Warming |
| --- | --- | --- | --- | --- | --- | --- | --- | --- |
| Nitrososphaeria | Nitrososphaerota | 52.33 | 53.07 | 53.46 | 50.21 | 54.39 | 52.61 | 50.24 |
| Methanomicrobia | Euryarchaeota | 2.56 | 2.44 | 2.38 | 3.01 | 1.89 | 2.83 | 2.89 |
| Halobacteria | Euryarchaeota | 1.95 | 2.41 | 1.67 | 1.87 | 1.54 | 1.99 | 2.21 |
| Thermoplasmata | Candidatus Thermoplasmatota | 1.71 | 1.46 | 1.73 | 2.04 | 1.19 | 1.96 | 1.99 |
| Methanobacteria | Euryarchaeota | 0.08 | 0.06 | 0.05 | 0.10 | 0.03 | 0.14 | 0.13 |
| Theionarchaea | Euryarchaeota | 0.03 | 0.01 | 0.03 | 0.01 | 0.02 | 0.01 | 0.13 |
| Others | Others | 41.34 | 40.56 | 40.67 | 42.76 | 40.94 | 40.46 | 42.41 |

**Table S5. Effects of experimental treatments on the relative abundance of taxonomic affiliations of transcripts associated with bacteria.** P, W, and P*W represented altered precipitation, warming, and the interaction of altered precipitation and warming, respectively. Coefficients of LMMs (relative abundance ~ altered precipitation*warming + (1|block)) represented the treatment effect sizes. Type II Wald chi-square tests were used to determine the statistical significance. Significant effects (*P* < 0.1) were bolded. R^2^_m_ and R^2^_c_ represented the marginal and conditional coefficients of determination for LMMs, respectively.

| Phylum/Class | Treatment | Coefficient | Standard error | t | Chi-square | df | *P* | R^2^_m_ | R^2^_c_ |
| --- | --- | --- | --- | --- | --- | --- | --- | --- | --- |
| **Actinomycetota** | **P** | **-0.06** | **0.48** | **-0.12** | **3.61** | **1** | **0.057** | 0.18 | 0.36 |
| Actinomycetota | W | 0.43 | 0.29 | 1.46 | 1.93 | 1 | 0.165 |  |  |
| **Actinomycetota** | **P*W** | **-1.27** | **0.70** | **-1.83** | **3.34** | **1** | **0.068** |  |  |
| **Acidobacteriota** | **P** | **0.67** | **0.46** | **1.44** | **3.19** | **1** | **0.074** | 0.09 | 0.44 |
| Acidobacteriota | W | -0.41 | 0.29 | -1.43 | 2.06 | 1 | 0.151 |  |  |
| Acidobacteriota | P*W | -0.14 | 0.68 | -0.21 | 0.05 | 1 | 0.830 |  |  |
| **Alphaproteobacteria** | **P** | **-0.07** | **0.49** | **-0.14** | **4.00** | **1** | **0.046** | 0.18 | 0.34 |
| Alphaproteobacteria | W | 0.32 | 0.30 | 1.07 | 0.99 | 1 | 0.321 |  |  |
| **Alphaproteobacteria** | **P*W** | **-1.35** | **0.71** | **-1.90** | **3.61** | **1** | **0.057** |  |  |
| Gammaproteobacteria | P | -0.60 | 0.57 | -1.04 | 1.57 | 1 | 0.211 | 0.10 | 0.10 |
| Gammaproteobacteria | W | -0.46 | 0.35 | -1.31 | 1.70 | 1 | 0.193 |  |  |
| Gammaproteobacteria | P*W | 0.16 | 0.83 | 0.20 | 0.04 | 1 | 0.845 |  |  |
| **Betaproteobacteria** | **P** | **0.87** | **0.54** | **1.60** | **5.91** | **1** | **0.015** | 0.18 | 0.18 |
| Betaproteobacteria | W | -0.35 | 0.33 | -1.05 | 1.09 | 1 | 0.296 |  |  |
| Betaproteobacteria | P*W | 0.18 | 0.79 | 0.23 | 0.05 | 1 | 0.820 |  |  |
| Chloroflexota | P | 0.17 | 0.43 | 0.39 | 1.47 | 1 | 0.226 | 0.14 | 0.50 |
| **Chloroflexota** | **W** | **0.53** | **0.26** | **2.01** | **3.76** | **1** | **0.053** |  |  |
| **Chloroflexota** | **P*W** | **-1.16** | **0.63** | **-1.85** | **3.43** | **1** | **0.064** |  |  |
| **Verrucomicrobiota** | **P** | **0.83** | **0.50** | **1.67** | **11.65** | **1** | **6.414E-04** | 0.30 | 0.30 |
| Verrucomicrobiota | W | -0.21 | 0.31 | -0.69 | 0.41 | 1 | 0.520 |  |  |
| Verrucomicrobiota | P*W | 0.84 | 0.72 | 1.16 | 1.35 | 1 | 0.245 |  |  |
| Candidatus Rokubacteria | P | 0.59 | 0.57 | 1.03 | 1.23 | 1 | 0.266 | 0.05 | 0.12 |
| Candidatus Rokubacteria | W | -0.27 | 0.35 | -0.78 | 0.63 | 1 | 0.429 |  |  |
| Candidatus Rokubacteria | P*W | -0.27 | 0.83 | -0.33 | 0.11 | 1 | 0.739 |  |  |
| **Planctomycetota** | **P** | **0.65** | **0.55** | **1.19** | **5.33** | **1** | **0.021** | 0.16 | 0.16 |
| Planctomycetota | W | -0.22 | 0.34 | -0.65 | 0.39 | 1 | 0.532 |  |  |
| Planctomycetota | P*W | 0.56 | 0.80 | 0.70 | 0.49 | 1 | 0.484 |  |  |

**Table S6. Effects of experimental treatments on the relative abundance of taxonomic affiliations of transcripts associated with methanotrophs and methanogens.** P, W, and P*W represented altered precipitation, warming, and the interaction of altered precipitation and warming, respectively. Coefficients of LMMs (relative abundance ~ altered precipitation*warming + (1|block)) represented the treatment effect sizes. Type II Wald chi-square tests were used to determine the statistical significance. Significant effects (*P* < 0.1) were bolded. R^2^_m_ and R^2^_c_ represented the marginal and conditional coefficients of determination for LMMs, respectively.

| Microbe | Treatment | Coefficient | Standard error | t | Chi-square | df | *P* | R^2^_m_ | R^2^_c_ |
| --- | --- | --- | --- | --- | --- | --- | --- | --- | --- |
| Methanotrophic bacteria | P | 1.03 | 0.46 | 2.26 | 1.78 | 1 | 0.182 | 0.10 | 0.44 |
| Methanotrophic bacteria | W | -0.14 | 0.28 | -0.49 | 0.32 | 1 | 0.570 |  |  |
| **Methanotrophic bacteria** | **P*W** | **-1.25** | **0.66** | **-1.88** | **3.52** | **1** | **0.061** |  |  |
| Methanotrophic archaea | P | 0.31 | 0.60 | 0.51 | 0.29 | 1 | 0.588 | 0.01 | 0.01 |
| Methanotrophic archaea | W | -0.03 | 0.37 | -0.08 | 0.01 | 1 | 0.929 |  |  |
| Methanotrophic archaea | P*W | -0.16 | 0.87 | -0.18 | 0.03 | 1 | 0.859 |  |  |
| **Methanogenic archaea** | **P** | **1.10** | **0.48** | **2.30** | **3.89** | **1** | **0.048** | 0.24 | 0.37 |
| **Methanogenic archaea** | **W** | **-0.74** | **0.29** | **-2.52** | **6.64** | **1** | **9.99E-03** |  |  |
| Methanogenic archaea | P*W | -0.88 | 0.69 | -1.27 | 1.61 | 1 | 0.205 |  |  |

**Table S7. Effect of experimental treatments on the relative abundance of taxonomic affiliations of methanotrophic transcripts**. P, W, and P*W represented altered precipitation, warming, and the interaction of altered precipitation and warming, respectively. Coefficients of LMMs (relative abundance ~ altered precipitation*warming + (1|block)) represented the treatment effect sizes. Type II Wald chi-square tests were used to determine the statistical significance. Significant effects (*P* < 0.1) were bolded. R^2^_m_ and R^2^_c_ represented the marginal and conditional coefficients of determination for LMMs, respectively.

| Methanotroph | Classification | Treatment | Coefficient | Standard error | t | Chi-square | df | *P* | R^2^_m_ | R^2^_c_ |
| --- | --- | --- | --- | --- | --- | --- | --- | --- | --- | --- |
| Archaea | Methanophagales | P | 0.41 | 0.60 | 0.69 | 0.50 | 1 | 0.480 | 0.02 | 0.02 |
| Archaea | Methanophagales | W | -0.13 | 0.37 | -0.35 | 0.13 | 1 | 0.715 |  |  |
| Archaea | Methanophagales | P*W | -0.22 | 0.87 | -0.26 | 0.07 | 1 | 0.797 |  |  |
| Archaea | Candidatus Methanoperedens | P | 0.05 | 0.57 | 0.08 | 0.01 | 1 | 0.928 | 0.00 | 0.13 |
| Archaea | Candidatus Methanoperedens | W | 0.12 | 0.35 | 0.34 | 0.12 | 1 | 0.734 |  |  |
| Archaea | Candidatus Methanoperedens | P*W | -0.02 | 0.83 | -0.03 | 0.00 | 1 | 0.978 |  |  |
| Bacteria | Crenothrix | P | 0.59 | 0.58 | 1.01 | 2.68 | 1 | 0.102 | 0.09 | 0.09 |
| Bacteria | Crenothrix | W | -0.16 | 0.35 | -0.46 | 0.21 | 1 | 0.650 |  |  |
| Bacteria | Crenothrix | P*W | 0.21 | 0.84 | 0.24 | 0.06 | 1 | 0.807 |  |  |
| Bacteria | Methylacidiphilum | P | -0.65 | 0.57 | -1.13 | 0.65 | 1 | 0.419 | 0.04 | 0.12 |
| Bacteria | Methylacidiphilum | W | 0.15 | 0.35 | 0.44 | 0.22 | 1 | 0.637 |  |  |
| Bacteria | Methylacidiphilum | P*W | 0.66 | 0.83 | 0.79 | 0.63 | 1 | 0.428 |  |  |
| Bacteria | Methylobacter | P | 0.70 | 0.57 | 1.22 | 2.69 | 1 | 0.101 | 0.10 | 0.10 |
| Bacteria | Methylobacter | W | -0.30 | 0.35 | -0.85 | 0.73 | 1 | 0.393 |  |  |
| Bacteria | Methylobacter | P*W | -0.04 | 0.83 | -0.05 | 0.00 | 1 | 0.957 |  |  |
| Bacteria | Methylocaldum | P | 0.56 | 0.48 | 1.17 | 0.00 | 1 | 0.996 | 0.06 | 0.39 |
| Bacteria | Methylocaldum | W | -0.10 | 0.29 | -0.36 | 0.18 | 1 | 0.671 |  |  |
| **Bacteria** | **Methylocaldum** | **P*W** | **-1.17** | **0.69** | **-1.69** | **2.86** | **1** | **0.091** |  |  |
| Bacteria | Methylocapsa | P | 0.52 | 0.60 | 0.87 | 0.39 | 1 | 0.535 | 0.04 | 0.04 |
| Bacteria | Methylocapsa | W | 0.23 | 0.37 | 0.63 | 0.37 | 1 | 0.545 |  |  |
| Bacteria | Methylocapsa | P*W | -0.53 | 0.86 | -0.61 | 0.37 | 1 | 0.541 |  |  |
| Bacteria | Methylocella | P | 0.47 | 0.50 | 0.95 | 0.67 | 1 | 0.413 | 0.02 | 0.36 |
| Bacteria | Methylocella | W | -0.11 | 0.31 | -0.34 | 0.13 | 1 | 0.715 |  |  |
| Bacteria | Methylocella | P*W | -0.38 | 0.72 | -0.52 | 0.27 | 1 | 0.600 |  |  |
| **Bacteria** | **Methylococcus** | **P** | **0.47** | **0.35** | **1.34** | **7.70** | **1** | **0.006** | 0.10 | 0.68 |
| Bacteria | Methylococcus | W | -0.22 | 0.21 | -1.05 | 1.03 | 1 | 0.310 |  |  |
| Bacteria | Methylococcus | P*W | 0.50 | 0.51 | 0.99 | 0.97 | 1 | 0.324 |  |  |
| **Bacteria** | **Methylocystis** | **P** | **-0.19** | **0.46** | **-0.41** | **3.46** | **1** | **0.063** | 0.12 | 0.40 |
| Bacteria | Methylocystis | W | 0.29 | 0.29 | 1.00 | 0.90 | 1 | 0.343 |  |  |
| Bacteria | Methylocystis | P*W | -0.92 | 0.68 | -1.36 | 1.85 | 1 | 0.174 |  |  |
| Bacteria | Methylohalobius | P | -0.95 | 0.50 | -1.90 | 0.95 | 1 | 0.329 | 0.09 | 0.33 |
| Bacteria | Methylohalobius | W | -0.19 | 0.31 | -0.60 | 0.29 | 1 | 0.592 |  |  |
| **Bacteria** | **Methylohalobius** | **P*W** | **1.26** | **0.73** | **1.74** | **3.02** | **1** | **0.082** |  |  |
| **Bacteria** | **Methylomicrobium** | **P** | **0.75** | **0.45** | **1.66** | **5.30** | **1** | **0.021** | 0.12 | 0.45 |
| Bacteria | Methylomicrobium | W | -0.37 | 0.28 | -1.35 | 1.83 | 1 | 0.177 |  |  |
| Bacteria | Methylomicrobium | P*W | 0.01 | 0.65 | 0.01 | 0.00 | 1 | 0.991 |  |  |
| Bacteria | Methylomonas | P | 0.35 | 0.55 | 0.63 | 2.03 | 1 | 0.154 | 0.09 | 0.17 |
| Bacteria | Methylomonas | W | 0.30 | 0.34 | 0.88 | 0.82 | 1 | 0.366 |  |  |
| Bacteria | Methylomonas | P*W | 0.47 | 0.80 | 0.58 | 0.34 | 1 | 0.561 |  |  |
| Bacteria | Methylosarcina | P | 1.11 | 0.53 | 2.08 | 0.68 | 1 | 0.411 | 0.20 | 0.22 |
| Bacteria | Methylosarcina | W | -0.49 | 0.33 | -1.51 | 2.55 | 1 | 0.110 |  |  |
| **Bacteria** | **Methylosarcina** | **P*W** | **-1.66** | **0.77** | **-2.16** | **4.65** | **1** | **0.031** |  |  |
| Bacteria | Methylosinus | P | 0.85 | 0.55 | 1.55 | 0.02 | 1 | 0.902 | 0.16 | 0.16 |
| Bacteria | Methylosinus | W | -0.18 | 0.34 | -0.54 | 0.40 | 1 | 0.528 |  |  |
| **Bacteria** | **Methylosinus** | **P*W** | **-1.89** | **0.80** | **-2.37** | **5.61** | **1** | **0.018** |  |  |

**Table S8. Effect of experimental treatments on the relative abundance of transcripts associated with CAZy.** P, W, and P*W represented altered precipitation, warming, and the interaction of altered precipitation and warming, respectively. Coefficients of LMMs (relative abundance ~ altered precipitation*warming + (1|block)) represented the treatment effect sizes. Type II Wald chi-square tests were used to determine the statistical significance. Significant effects (*P* < 0.1) were bolded. R^2^_m_ and R^2^_c_ represented the marginal and conditional coefficients of determination for LMMs, respectively. AA, auxiliary activities; CBM, carbohydrate binding; CE, carbohydrate esterases; GH, glycoside hydrolases; GT, glycosyltransferases; PL, polysaccharide lyases.

| CAZyme | Treatment | Coefficient | Standard error | t | Chi-square | df | *P* | R^2^_m_ | R^2^_c_ |
| --- | --- | --- | --- | --- | --- | --- | --- | --- | --- |
| CAZy | P | 0.53 | 0.49 | 1.08 | 1.14 | 1 | 0.285 | 0.20 | 0.33 |
| CAZy | W | 0.33 | 0.30 | 1.09 | 0.97 | 1 | 0.324 |  |  |
| **CAZy** | **P*W** | **-1.92** | **0.71** | **-2.69** | **7.25** | **1** | **0.007** |  |  |
| CE | P | -0.06 | 0.55 | -0.11 | 1.25 | 1 | 0.263 | 0.07 | 0.18 |
| CE | W | 0.18 | 0.34 | 0.54 | 0.25 | 1 | 0.619 |  |  |
| CE | P*W | -0.81 | 0.79 | -1.02 | 1.04 | 1 | 0.308 |  |  |
| GH | P | 0.82 | 0.51 | 1.62 | 0.00 | 1 | 0.984 | 0.13 | 0.29 |
| GH | W | 0.14 | 0.31 | 0.45 | 0.13 | 1 | 0.719 |  |  |
| **GH** | **P*W** | **-1.75** | **0.74** | **-2.37** | **5.60** | **1** | **0.018** |  |  |
| **PL** | **P** | **-0.82** | **0.44** | **-1.85** | **8.27** | **1** | **0.004** | 0.14 | 0.47 |
| PL | W | 0.05 | 0.27 | 0.20 | 0.03 | 1 | 0.852 |  |  |
| PL | P*W | -0.22 | 0.65 | -0.35 | 0.12 | 1 | 0.729 |  |  |
| **GT** | **P** | **-0.07** | **0.46** | **-0.16** | **6.07** | **1** | **0.014** | 0.29 | 0.40 |
| **GT** | **W** | **0.58** | **0.28** | **2.06** | **3.89** | **1** | **0.049** |  |  |
| **GT** | **P*W** | **-1.57** | **0.67** | **-2.36** | **5.56** | **1** | **0.018** |  |  |
| **AA** | **P** | **-0.23** | **0.54** | **-0.42** | **3.53** | **1** | **0.060** | 0.18 | 0.19 |
| AA | W | 0.45 | 0.33 | 1.35 | 1.67 | 1 | 0.196 |  |  |
| AA | P*W | -1.06 | 0.78 | -1.35 | 1.83 | 1 | 0.176 |  |  |
| **CBM** | **P** | **-0.45** | **0.31** | **-1.46** | **10.15** | **1** | **0.001** | 0.12 | 0.73 |
| CBM | W | 0.28 | 0.19 | 1.46 | 2.00 | 1 | 0.158 |  |  |
| CBM | P*W | -0.56 | 0.45 | -1.25 | 1.56 | 1 | 0.212 |  |  |

**Table S9. The relative abundance of transcripts associated with methane metabolism across drought, ambient, wet, drought and warming, warming, as well as wet and warming plots.** Methane metabolism included methanotrophy (4 gene families) and methanogenesis (14 gene families), which was divided into methylotrophic methanogenesis (2 gene families), aceticlastic methanogenesis (3 gene families), and hydrogenotrophic methanogenesis (6 gene families) based on the substrates and core methanogenesis (3 gene families).

| KO/ Transcript | Transcript | Pathway | Drought | Ambient | Wet | Drought + Warming | Warming | Wet + Warming |
| --- | --- | --- | --- | --- | --- | --- | --- | --- |
| K23995 | *xoxF* | Methanotrophy | 71.78 | 64.93 | 59.10 | 68.78 | 63.11 | 71.88 |
| K10946 | *pmoC-amoC* | Methanotrophy | 88.23 | 97.05 | 86.47 | 106.10 | 71.55 | 75.99 |
| K10945 | *pmoB-amoB* | Methanotrophy | 39.95 | 43.48 | 41.31 | 55.81 | 33.89 | 35.16 |
| K10944 | *pmoA-amoA* | Methanotrophy | 21.13 | 23.97 | 21.58 | 25.25 | 16.76 | 22.28 |
| K14084 | *mttC* | Methylotrophic | 0.24 | 0.72 | 0.52 | 0.62 | 0.41 | 0.28 |
| K14083 | *mttB* | Methylotrophic | 26.81 | 36.17 | 22.63 | 43.82 | 30.70 | 20.96 |
| K01895 | *ACSS1_2* | Aceticlastic | 19.43 | 22.18 | 18.82 | 33.43 | 31.57 | 15.54 |
| K00925 | *ackA* | Aceticlastic | 13.15 | 18.10 | 13.29 | 13.73 | 12.53 | 12.14 |
| K00625 | *pta* | Aceticlastic | 9.42 | 10.96 | 7.11 | 7.51 | 6.95 | 7.95 |
| K01499 | *mch* | Hydrogenotrophic | 0.00 | 0.06 | 0.00 | 0.00 | 2.58 | 0.00 |
| K00672 | *ftr* | Hydrogenotrophic | 0.50 | 0.73 | 0.30 | 0.83 | 3.52 | 0.33 |
| K00320 | *mer* | Hydrogenotrophic | 1.56 | 2.12 | 1.29 | 1.83 | 2.11 | 0.68 |
| K00202 | *fwdC* | Hydrogenotrophic | 0.00 | 0.00 | 0.00 | 0.00 | 1.10 | 0.00 |
| K00201 | *fwdB* | Hydrogenotrophic | 1.81 | 1.52 | 1.64 | 1.89 | 4.39 | 1.79 |
| K00200 | *fwdA* | Hydrogenotrophic | 0.00 | 0.03 | 0.05 | 0.00 | 4.63 | 0.00 |
| *mcrC* | *mcrC* | Core methanogenesis | 11.12 | 11.68 | 8.25 | 24.13 | 18.25 | 7.50 |
| *mcrB* | *mcrB* | Core methanogenesis | 1.26 | 2.00 | 0.70 | 1.11 | 0.77 | 0.97 |
| *mcrA* | *mcrA* | Core methanogenesis | 2173 | 1741 | 2028 | 1802 | 1783 | 1858 |

**Table S10. Effect of experimental treatments on the relative abundance of transcripts associated with methane metabolism.** P, W, and P*W represented altered precipitation, warming, and the interaction of altered precipitation and warming, respectively. Methane metabolism included methanotrophy (4 gene families) and methanogenesis (14 gene families), which was divided into methylotrophic methanogenesis (2 gene families), aceticlastic methanogenesis (3 gene families), and hydrogenotrophic methanogenesis (6 gene families) based on the substrates and core methanogenesis (3 gene families). Coefficients represented regression coefficients of LMMs (relative abundance ~ altered precipitation*warming + (1|block)), and *P* represented the statistical significance determined by Type II Wald chi-square tests. Significant effects (*P* < 0.1) were bolded. R^2^_m_ and R^2^_c_ represented the marginal and conditional coefficients of determination for LMMs, respectively.

| KO/ Transcript | Transcript | Pathway | Treatment | Coefficient | Standard error | t | Chi-square | df | *P* | R^2^_m_ | R^2^_c_ |
| --- | --- | --- | --- | --- | --- | --- | --- | --- | --- | --- | --- |
| K23995 | *xoxF* | Methanotrophy | P | -0.33 | 0.32 | -1.06 | 0.11 | 1 | 0.737 | 0.01 | 0.73 |
| K23995 | *xoxF* | Methanotrophy | W | 0.07 | 0.19 | 0.35 | 0.16 | 1 | 0.689 |  |  |
| K23995 | *xoxF* | Methanotrophy | P*W | 0.54 | 0.46 | 1.18 | 1.39 | 1 | 0.238 |  |  |
| K10946 | *pmoC-amoC* | Methanotrophy | P | -0.06 | 0.52 | -0.12 | 1.43 | 1 | 0.232 | 0.07 | 0.26 |
| K10946 | *pmoC-amoC* | Methanotrophy | W | -0.17 | 0.32 | -0.53 | 0.33 | 1 | 0.566 |  |  |
| K10946 | *pmoC-amoC* | Methanotrophy | P*W | -0.82 | 0.76 | -1.09 | 1.19 | 1 | 0.275 |  |  |
| **K10945** | ***pmoB-amoB*** | **Methanotrophy** | **P** | **0.10** | **0.50** | **0.21** | **3.01** | **1** | **0.083** | 0.17 | 0.30 |
| K10945 | *pmoB-amoB* | Methanotrophy | W | 0.04 | 0.31 | 0.12 | 0.00 | 1 | 0.974 |  |  |
| **K10945** | ***pmoB-amoB*** | **Methanotrophy** | **P*W** | **-1.55** | **0.73** | **-2.13** | **4.53** | **1** | **0.033** |  |  |
| K10944 | *pmoA-amoA* | Methanotrophy | P | 0.06 | 0.48 | 0.13 | 0.05 | 1 | 0.820 | 0.01 | 0.39 |
| K10944 | *pmoA-amoA* | Methanotrophy | W | -0.07 | 0.29 | -0.22 | 0.06 | 1 | 0.809 |  |  |
| K10944 | *pmoA-amoA* | Methanotrophy | P*W | -0.30 | 0.69 | -0.43 | 0.19 | 1 | 0.664 |  |  |
| K14084 | *mttC* | Methylotrophic | P | 0.54 | 0.59 | 0.92 | 0.01 | 1 | 0.940 | 0.06 | 0.06 |
| K14084 | *mttC* | Methylotrophic | W | -0.05 | 0.36 | -0.15 | 0.04 | 1 | 0.841 |  |  |
| K14084 | *mttC* | Methylotrophic | P*W | -1.19 | 0.85 | -1.41 | 1.98 | 1 | 0.160 |  |  |
| **K14083** | ***mttB*** | **Methylotrophic** | **P** | **-0.23** | **0.40** | **-0.57** | **4.74** | **1** | **0.029** | 0.11 | 0.56 |
| K14083 | *mttB* | Methylotrophic | W | 0.23 | 0.25 | 0.92 | 0.75 | 1 | 0.386 |  |  |
| K14083 | *mttB* | Methylotrophic | P*W | -0.86 | 0.58 | -1.48 | 2.18 | 1 | 0.140 |  |  |
| **K01895** | ***ACSS1_2*** | **Aceticlastic** | **P** | **-0.04** | **0.47** | **-0.09** | **3.79** | **1** | **0.052** | 0.20 | 0.39 |
| **K01895** | ***ACSS1_2*** | **Aceticlastic** | **W** | **0.50** | **0.29** | **1.73** | **2.73** | **1** | **0.098** |  |  |
| **K01895** | ***ACSS1_2*** | **Aceticlastic** | **P*W** | **-1.31** | **0.68** | **-1.91** | **3.67** | **1** | **0.056** |  |  |
| K00925 | *ackA* | Aceticlastic | P | 0.02 | 0.55 | 0.04 | 0.04 | 1 | 0.835 | 0.02 | 0.19 |
| K00925 | *ackA* | Aceticlastic | W | -0.26 | 0.33 | -0.77 | 0.61 | 1 | 0.436 |  |  |
| K00925 | *ackA* | Aceticlastic | P*W | -0.22 | 0.79 | -0.27 | 0.08 | 1 | 0.784 |  |  |
| K00625 | *pta* | Aceticlastic | P | -0.38 | 0.43 | -0.89 | 0.08 | 1 | 0.774 | 0.03 | 0.53 |
| K00625 | *pta* | Aceticlastic | W | -0.24 | 0.26 | -0.93 | 0.79 | 1 | 0.375 |  |  |
| K00625 | *pta* | Aceticlastic | P*W | 0.61 | 0.62 | 0.99 | 0.97 | 1 | 0.324 |  |  |
| K01499 | *mch* | Hydrogenotrophic | P | 0.00 | 0.59 | 0.00 | 0.01 | 1 | 0.923 | 0.03 | 0.05 |
| K01499 | *mch* | Hydrogenotrophic | W | 0.35 | 0.36 | 0.96 | 0.92 | 1 | 0.337 |  |  |
| K01499 | *mch* | Hydrogenotrophic | P*W | -0.09 | 0.86 | -0.10 | 0.01 | 1 | 0.920 |  |  |
| K00672 | *ftr* | Hydrogenotrophic | P | -0.08 | 0.58 | -0.14 | 0.18 | 1 | 0.671 | 0.04 | 0.08 |
| K00672 | *ftr* | Hydrogenotrophic | W | 0.40 | 0.36 | 1.12 | 1.23 | 1 | 0.268 |  |  |
| K00672 | *ftr* | Hydrogenotrophic | P*W | -0.21 | 0.85 | -0.25 | 0.06 | 1 | 0.803 |  |  |
| K00320 | *mer* | Hydrogenotrophic | P | -0.22 | 0.58 | -0.38 | 1.92 | 1 | 0.165 | 0.08 | 0.08 |
| K00320 | *mer* | Hydrogenotrophic | W | -0.06 | 0.36 | -0.17 | 0.04 | 1 | 0.840 |  |  |
| K00320 | *mer* | Hydrogenotrophic | P*W | -0.75 | 0.84 | -0.90 | 0.81 | 1 | 0.369 |  |  |
| K00202 | *fwdC* | Hydrogenotrophic | P | 0.00 | 0.59 | 0.00 | 0.01 | 1 | 0.921 | 0.03 | 0.06 |
| K00202 | *fwdC* | Hydrogenotrophic | W | 0.36 | 0.36 | 0.98 | 0.96 | 1 | 0.326 |  |  |
| K00202 | *fwdC* | Hydrogenotrophic | P*W | -0.09 | 0.85 | -0.10 | 0.01 | 1 | 0.917 |  |  |
| K00201 | *fwdB* | Hydrogenotrophic | P | -0.08 | 0.59 | -0.13 | 0.05 | 1 | 0.828 | 0.05 | 0.05 |
| K00201 | *fwdB* | Hydrogenotrophic | W | 0.44 | 0.36 | 1.20 | 1.44 | 1 | 0.231 |  |  |
| K00201 | *fwdB* | Hydrogenotrophic | P*W | -0.04 | 0.86 | -0.04 | 0.00 | 1 | 0.967 |  |  |
| K00200 | *fwdA* | Hydrogenotrophic | P | 0.01 | 0.59 | 0.02 | 0.01 | 1 | 0.936 | 0.03 | 0.05 |
| K00200 | *fwdA* | Hydrogenotrophic | W | 0.35 | 0.36 | 0.96 | 0.92 | 1 | 0.337 |  |  |
| K00200 | *fwdA* | Hydrogenotrophic | P*W | -0.10 | 0.86 | -0.12 | 0.01 | 1 | 0.907 |  |  |
| ***mcrC*** | ***mcrC*** | **Core methanogenesis** | **P** | **-0.22** | **0.54** | **-0.40** | **3.36** | **1** | **0.067** | 0.18 | 0.18 |
| *mcrC* | *mcrC* | Core methanogenesis | W | 0.49 | 0.33 | 1.45 | 1.97 | 1 | 0.160 |  |  |
| *mcrC* | *mcrC* | Core methanogenesis | P*W | -1.05 | 0.79 | -1.34 | 1.79 | 1 | 0.181 |  |  |
| *mcrB* | *mcrB* | Core methanogenesis | P | -0.47 | 0.58 | -0.82 | 0.47 | 1 | 0.493 | 0.03 | 0.10 |
| *mcrB* | *mcrB* | Core methanogenesis | W | -0.24 | 0.35 | -0.68 | 0.44 | 1 | 0.506 |  |  |
| *mcrB* | *mcrB* | Core methanogenesis | P*W | 0.40 | 0.84 | 0.47 | 0.23 | 1 | 0.635 |  |  |
| *mcrA* | *mcrA* | Core methanogenesis | P | -0.21 | 0.41 | -0.50 | 0.00 | 1 | 0.995 | 0.03 | 0.56 |
| *mcrA* | *mcrA* | Core methanogenesis | W | -0.28 | 0.25 | -1.13 | 1.21 | 1 | 0.272 |  |  |
| *mcrA* | *mcrA* | Core methanogenesis | P*W | 0.44 | 0.60 | 0.74 | 0.55 | 1 | 0.459 |  |  |

**Table S11. Effect of experimental treatments on the relative abundance of transcripts related to transporters and catabolism of sugars and acids.** P, W, and P*W represented altered precipitation, warming, and the interaction of altered precipitation and warming, respectively. Coefficients represented regression coefficients of LMMs (relative abundance ~ altered precipitation*warming + (1|block)), and *P* represented the statistical significance determined by Type II Wald chi-square tests. Significant effects (*P* < 0.1) were bolded. R^2^_m_ and R^2^_c_ represented the marginal and conditional coefficients of determination for LMMs, respectively.

| Metabolism | Treatment | Coefficient | Standard error | t | Chi-square | df | *P* | R^2^_m_ | R^2^_c_ |
| --- | --- | --- | --- | --- | --- | --- | --- | --- | --- |
| **Transporter** | **P** | **-0.28** | **0.30** | **-0.92** | **4.29** | **1** | **0.038** | 0.04 | 0.76 |
| Transporter | W | 0.16 | 0.18 | 0.85 | 0.67 | 1 | 0.413 |  |  |
| Transporter | P*W | -0.37 | 0.43 | -0.84 | 0.71 | 1 | 0.398 |  |  |
| **Sugar transporter** | **P** | **-0.43** | **0.37** | **-1.16** | **7.06** | **1** | **0.008** | 0.12 | 0.62 |
| Sugar transporter | W | 0.30 | 0.23 | 1.33 | 1.66 | 1 | 0.197 |  |  |
| Sugar transporter | P*W | -0.61 | 0.54 | -1.13 | 1.27 | 1 | 0.261 |  |  |
| Acid transporter | P | -0.12 | 0.27 | -0.46 | 0.97 | 1 | 0.324 | 0.01 | 0.81 |
| Acid transporter | W | 0.03 | 0.16 | 0.21 | 0.04 | 1 | 0.846 |  |  |
| Acid transporter | P*W | -0.14 | 0.39 | -0.37 | 0.14 | 1 | 0.711 |  |  |
| **SAR-Transporter** | **P** | **-0.53** | **0.43** | **-1.25** | **9.27** | **1** | **0.002** | 0.24 | 0.49 |
| **SAR-Transporter** | **W** | **0.53** | **0.26** | **2.03** | **3.90** | **1** | **0.048** |  |  |
| SAR-Transporter | P*W | -0.86 | 0.62 | -1.39 | 1.94 | 1 | 0.164 |  |  |
| **Catabolism** | **P** | **-0.2** | **0.44** | **-0.46** | **5.35** | **1** | **0.021** | 0.16 | 0.47 |
| Catabolism | W | 0.25 | 0.27 | 0.94 | 0.77 | 1 | 0.381 |  |  |
| **Catabolism** | **P*W** | **-1.12** | **0.63** | **-1.76** | **3.11** | **1** | **0.078** |  |  |
| **Sugar catabolism** | **P** | **-0.25** | **0.41** | **-0.62** | **6.85** | **1** | **0.009** | 0.169 | 0.540 |
| Sugar catabolism | W | 0.28 | 0.25 | 1.13 | 1.11 | 1 | 0.292 |  |  |
| **Sugar catabolism** | **P*W** | **-1.1** | **0.59** | **-1.86** | **3.47** | **1** | **0.063** |  |  |
| **Acid catabolism** | **P** | **-0.18** | **0.44** | **-0.42** | **4.95** | **1** | **0.026** | 0.148 | 0.469 |
| Acid catabolism | W | 0.24 | 0.27 | 0.89 | 0.68 | 1 | 0.408 |  |  |
| **Acid catabolism** | **P*W** | **-1.1** | **0.63** | **-1.73** | **3.01** | **1** | **0.083** |  |  |
| **SAR-Catabolism** | **P** | **-0.36** | **0.41** | **-0.87** | **5.67** | **1** | **0.017** | 0.118 | 0.569 |
| SAR-Catabolism | W | 0.3 | 0.25 | 1.21 | 1.35 | 1 | 0.245 |  |  |
| SAR-Catabolism | P*W | -0.74 | 0.59 | -1.25 | 1.55 | 1 | 0.213 |  |  |

**Table S12. Effect of experimental treatments on the relative abundance of taxonomic affiliations of transcripts associated with sugar and acid transporters.** P, W, and P*W represented altered precipitation, warming, and the interaction of altered precipitation and warming, respectively. Coefficients of LMMs (taxonomy ~ altered precipitation*warming + (1|block)) represented the treatment effect sizes. Type II Wald chi-square tests were used to determine the statistical significance. Significant effects (*P* < 0.1) were bolded. R^2^_m_ and R^2^_c_ represented the marginal and conditional coefficients of determination for LMMs, respectively.

| Taxonomy | Treatment | Coefficient | Standard error | t | Chi-square | df | *P* | R^2^_m_ | R^2^_c_ |
| --- | --- | --- | --- | --- | --- | --- | --- | --- | --- |
| **Actinomycetota** | **P** | **-0.43** | **0.41** | **-1.04** | **7.93** | **1** | **0.005** | 0.17 | 0.53 |
| Actinomycetota | W | 0.26 | 0.25 | 1.04 | 0.97 | 1 | 0.324 |  |  |
| Actinomycetota | P*W | -0.88 | 0.60 | -1.46 | 2.14 | 1 | 0.144 |  |  |
| Acidobacteriota | P | -0.17 | 0.48 | -0.36 | 0.96 | 1 | 0.328 | 0.03 | 0.37 |
| Acidobacteriota | W | 0.22 | 0.30 | 0.74 | 0.52 | 1 | 0.469 |  |  |
| Acidobacteriota | P*W | -0.36 | 0.70 | -0.51 | 0.26 | 1 | 0.608 |  |  |
| **Alphaproteobacteria** | **P** | **-0.30** | **0.32** | **-0.95** | **3.60** | **1** | **0.058** | 0.05 | 0.73 |
| Alphaproteobacteria | W | 0.24 | 0.19 | 1.25 | 1.50 | 1 | 0.221 |  |  |
| Alphaproteobacteria | P*W | -0.28 | 0.46 | -0.62 | 0.38 | 1 | 0.537 |  |  |
| Gammaproteobacteria | P | -0.47 | 0.38 | -1.25 | 2.01 | 1 | 0.156 | 0.04 | 0.64 |
| Gammaproteobacteria | W | -0.23 | 0.23 | -0.98 | 0.94 | 1 | 0.332 |  |  |
| Gammaproteobacteria | P*W | 0.18 | 0.55 | 0.32 | 0.10 | 1 | 0.746 |  |  |
| **Betaproteobacteria** | **P** | **0.55** | **0.40** | **1.38** | **5.99** | **1** | **0.014** | 0.12 | 0.56 |
| Betaproteobacteria | W | -0.36 | 0.25 | -1.46 | 2.06 | 1 | 0.151 |  |  |
| Betaproteobacteria | P*W | 0.34 | 0.58 | 0.58 | 0.33 | 1 | 0.564 |  |  |
| Chloroflexota | P | -0.38 | 0.31 | -1.22 | 0.91 | 1 | 0.340 | 0.06 | 0.74 |
| **Chloroflexota** | **W** | **0.46** | **0.19** | **2.38** | **5.81** | **1** | **0.016** |  |  |
| Chloroflexota | P*W | 0.35 | 0.46 | 0.76 | 0.58 | 1 | 0.446 |  |  |
| Verrucomicrobiota | P | 0.53 | 0.48 | 1.10 | 0.72 | 1 | 0.397 | 0.04 | 0.38 |
| Verrucomicrobiota | W | -0.26 | 0.30 | -0.88 | 0.82 | 1 | 0.364 |  |  |
| Verrucomicrobiota | P*W | -0.50 | 0.70 | -0.71 | 0.50 | 1 | 0.478 |  |  |
| Candidatus Rokubacteria | P | 0.22 | 0.60 | 0.37 | 0.25 | 1 | 0.618 | 0.01 | 0.01 |
| Candidatus Rokubacteria | W | -0.10 | 0.37 | -0.26 | 0.07 | 1 | 0.795 |  |  |
| Candidatus Rokubacteria | P*W | -0.01 | 0.87 | -0.01 | 0.00 | 1 | 0.994 |  |  |
| Planctomycetota | P | -0.43 | 0.45 | -0.95 | 0.32 | 1 | 0.570 | 0.02 | 0.45 |
| Planctomycetota | W | 0.07 | 0.28 | 0.26 | 0.08 | 1 | 0.771 |  |  |
| Planctomycetota | P*W | 0.51 | 0.66 | 0.78 | 0.61 | 1 | 0.436 |  |  |
| Myxococcota | P | 0.25 | 0.38 | 0.66 | 0.48 | 1 | 0.488 | 0.01 | 0.61 |
| Myxococcota | W | 0.08 | 0.24 | 0.33 | 0.11 | 1 | 0.745 |  |  |
| Myxococcota | P*W | -0.13 | 0.56 | -0.23 | 0.05 | 1 | 0.817 |  |  |
| Archaea | P | -0.23 | 0.53 | -0.43 | 2.63 | 1 | 0.105 | 0.13 | 0.22 |
| Archaea | W | -0.37 | 0.33 | -1.12 | 1.36 | 1 | 0.244 |  |  |
| Archaea | P*W | -0.83 | 0.77 | -1.08 | 1.16 | 1 | 0.281 |  |  |

**Table S13. Correlations between soil carbon fluxes and environmental variables and microbial carbon metabolism.** Environmental variables included soil temperature, soil moisture, and pH. Microbial carbon metabolism included carbohydrate metabolism, methane metabolism, and carbon substrate preferences. *r* represented the correlation coefficient determined by LMMs (flux/ratio ~ environmental variable/microbial carbon metabolism + (1|block)). Type II Wald chi-square tests were used to determine statistical significance (*P*). Significant correlations (*P* < 0.1) were bolded. CO_2_ represented the heterotrophic respiration flux. Methanogenesis included the core methanogenesis, methylotrophic methanogenesis, aceticlastic methanogenesis, and hydrogenotrophic methanogenesis. Carbohydrate metabolism included glycoside hydrolases (GH), glycosyltransferases (GT), polysaccharide lyases (PL), auxiliary activities (AA), carbohydrate esterases (CE), carbohydrate binding (CBM).

| Carbon flux/Ratio | Variable | *r* | R^2^ | Chi-square | df | *P* |
| --- | --- | --- | --- | --- | --- | --- |
| **CH_4_** | **Temperature** | **-0.56** | **0.31** | **4.94** | **1** | **0.026** |
| CH_4_ | Moisture | 0.43 | 0.19 | 2.54 | 1 | 0.111 |
| **CH_4_** | **pH** | **-0.58** | **0.34** | **10.24** | **1** | **0.001** |
| CH_4_ | CAZy | -0.29 | 0.08 | 1.33 | 1 | 0.248 |
| CH_4_ | AA | -0.22 | 0.05 | 0.99 | 1 | 0.320 |
| CH_4_ | CBM | -0.40 | 0.16 | 2.29 | 1 | 0.130 |
| CH_4_ | CE | -0.28 | 0.08 | 1.34 | 1 | 0.248 |
| CH_4_ | GH | -0.11 | 0.01 | 0.18 | 1 | 0.670 |
| **CH_4_** | **GT** | **-0.47** | **0.22** | **5.09** | **1** | **0.024** |
| **CH_4_** | **PL** | **-0.46** | **0.21** | **3.91** | **1** | **0.048** |
| CH_4_ | Sugar transporter | -0.24 | 0.06 | 0.71 | 1 | 0.400 |
| CH_4_ | Acid transporter | -0.12 | 0.02 | 0.14 | 1 | 0.705 |
| CH_4_ | SAR-Transporter | -0.26 | 0.07 | 1.48 | 1 | 0.223 |
| **CH_4_** | **Methanogenesis** | **0.43** | **0.19** | **4.10** | **1** | **0.043** |
| CH_4_ | Methanotrophy | 0.07 | 0.01 | 0.07 | 1 | 0.797 |
| CO_2_ | Temperature | 0.07 | 0.00 | 0.08 | 1 | 0.772 |
| CO_2_ | Moisture | 0.09 | 0.01 | 0.18 | 1 | 0.670 |
| CO_2_ | pH | 0.16 | 0.03 | 0.62 | 1 | 0.432 |
| CO_2_ | CAZy | 0.24 | 0.06 | 1.34 | 1 | 0.247 |
| CO_2_ | AA | 0.00 | 0.00 | 0.00 | 1 | 0.989 |
| CO_2_ | CBM | 0.31 | 0.10 | 2.15 | 1 | 0.143 |
| CO_2_ | CE | 0.08 | 0.01 | 0.12 | 1 | 0.727 |
| CO_2_ | GH | 0.15 | 0.02 | 0.47 | 1 | 0.493 |
| **CO_2_** | **GT** | **0.33** | **0.11** | **3.68** | **1** | **0.055** |
| CO_2_ | PL | 0.04 | 0.00 | 0.03 | 1 | 0.871 |
| CO_2_ | Sugar transporter | -0.05 | 0.00 | 0.03 | 1 | 0.856 |
| CO_2_ | Acid transporter | -0.02 | 0.00 | 0.00 | 1 | 0.962 |
| CO_2_ | SAR-Transporter | -0.14 | 0.02 | 0.45 | 1 | 0.503 |
| CO_2_ | Methanogenesis | -0.21 | 0.04 | 0.52 | 1 | 0.469 |
| CO_2_ | Methanotrophy | -0.39 | 0.15 | 2.09 | 1 | 0.148 |
| **CH_4_/CO_2_** | **Temperature** | **-0.43** | **0.19** | **4.97** | **1** | **0.026** |
| **CH_4_/CO_2_** | **Moisture** | **0.47** | **0.22** | **3.42** | **1** | **0.064** |
| **CH_4_/CO_2_** | **pH** | **-0.47** | **0.22** | **6.42** | **1** | **0.011** |
| CH_4_/CO_2_ | CAZy | -0.34 | 0.12 | 1.96 | 1 | 0.161 |
| CH_4_/CO_2_ | AA | -0.32 | 0.10 | 1.90 | 1 | 0.168 |
| **CH_4_/CO_2_** | **CBM** | **-0.46** | **0.21** | **3.95** | **1** | **0.047** |
| **CH_4_/CO_2_** | **CE** | **-0.40** | **0.16** | **2.90** | **1** | **0.089** |
| CH_4_/CO_2_ | GH | -0.12 | 0.02 | 0.23 | 1 | 0.633 |
| **CH_4_/CO_2_** | **GT** | **-0.42** | **0.17** | **3.14** | **1** | **0.076** |
| **CH_4_/CO_2_** | **PL** | **-0.61** | **0.37** | **8.87** | **1** | **0.003** |
| **CH_4_/CO_2_** | **Sugar transporter** | **-0.43** | **0.19** | **3.44** | **1** | **0.064** |
| CH_4_/CO_2_ | Acid transporter | -0.38 | 0.14 | 2.46 | 1 | 0.117 |
| **CH_4_/CO_2_** | **SAR-Transporter** | **-0.34** | **0.12** | **3.03** | **1** | **0.082** |
| **CH_4_/CO_2_** | **Methanogenesis** | **0.38** | **0.15** | **3.42** | **1** | **0.064** |
| CH_4_/CO_2_ | Methanotrophy | -0.29 | 0.08 | 1.36 | 1 | 0.244 |

**Table S14. Effects of environmental variables and microbial carbon metabolism on CH_4_/CO_2_.** Standardized regression coefficients of LMMs (CH_4_/CO_2_ ~ soil moisture + soil temperature + SAR-Transporter + CAZy + (1|block)) represented the effect sizes of environmental variables and microbial carbon metabolism on CH_4_/CO_2_ based on rescaled predictors. Type II Wald chi-square tests were used to determine the statistical significance. Significant effects (*P* < 0.1) were bolded. R^2^_m_ and R^2^_c_ represented the marginal and conditional coefficients of determination for LMMs, respectively.

| Predictor | Coefficient | Standard error | t | Chi-square | df | *P* | R^2^_m_ | R^2^_c_ |
| --- | --- | --- | --- | --- | --- | --- | --- | --- |
| Soil moiture | 0.18 | 0.28 | 0.62 | 0.39 | 1 | 0.532 | 0.25 | 0.78 |
| Soil temperature | -0.32 | 0.35 | -0.90 | 0.80 | 1 | 0.370 |  |  |
| CAZy | -0.13 | 0.45 | -0.29 | 0.08 | 1 | 0.772 |  |  |
| **SAR-Transporter** | **-0.52** | **0.25** | **-2.10** | **4.42** | **1** | **0.036** |  |  |

**References**

1. Jia J, Cao Z, Liu C, Zhang Z, Lin L, Wang Y, Haghipour N, Wacker L, Bao H, Dittmar T, Simpson M, Yang H, Crowther T, Eglinton T, He J, Feng X. 2019. Climate warming alters subsoil but not topsoil carbon dynamics in alpine grassland. Glob Change Biol 25:4383-4393.
